# Imidazole and Imidazolium Antibacterial Drugs Derived from Amino Acids

**DOI:** 10.3390/ph13120482

**Published:** 2020-12-21

**Authors:** Adriana Valls, Jose J. Andreu, Eva Falomir, Santiago V. Luis, Elena Atrián-Blasco, Scott G. Mitchell, Belén Altava

**Affiliations:** 1 Departamento de Química Inorgánica y Orgánica, Universitat Jaume I, Av. Sos Baynat s/n, 12071 Castellón, Spain; avalls@uji.es (A.V.); al314093@uji.es (J.J.A.); efalomir@uji.es (E.F.); luiss@uji.es (S.V.L.); 2 Instituto de Nanociencia y Materiales de Aragón (INMA), Consejo Superior de Investigaciones Científicas-Universidad de Zaragoza, 50009 Zaragoza, Spain; 3 CIBER de Bioingeniería, Biomateriales y Nanomedicina, Instituto de Salud Carlos III, 28029 Madrid, Spain

**Keywords:** imidazole and imidazolium salts, amino acid, antibacterial agents, aggregation, lipophilicity

## Abstract

The antibacterial activity of imidazole and imidazolium salts is highly dependent upon their lipophilicity, which can be tuned through the introduction of different hydrophobic substituents on the nitrogen atoms of the imidazole or imidazolium ring of the molecule. Taking this into consideration, we have synthesized and characterized a series of imidazole and imidazolium salts derived from *L*-valine and *L*-phenylalanine containing different hydrophobic groups and tested their antibacterial activity against two model bacterial strains, Gram-negative *E. coli* and Gram-positive *B. subtilis*. Importantly, the results demonstrate that the minimum bactericidal concentration (MBC) of these derivatives can be tuned to fall close to the cytotoxicity values in eukaryotic cell lines. The MBC value of one of these compounds toward *B. subtilis* was found to be lower than the IC_50_ cytotoxicity value for the control cell line, HEK-293. Furthermore, the aggregation behavior of these compounds has been studied in pure water, in cell culture media, and in mixtures thereof, in order to determine if the compounds formed self-assembled aggregates at their bioactive concentrations with the aim of determining whether the monomeric species were in fact responsible for the observed antibacterial activity. Overall, these results indicate that imidazole and imidazolium compounds derived from *L*-valine and *L*-phenylalanine—with different alkyl lengths in the amide substitution—can serve as potent antibacterial agents with low cytotoxicity to human cell lines.

## 1. Introduction

Aromatic heterocycles, particularly the imidazole ring, have been used in the last decades as structural skeletons to obtain different types of bioactive compounds with antibacterial, antifungal, anticancer, antiviral, antidiabetic, and other properties [1,2,3,4]. The search for new potent drug molecules derived from imidazole continues to be an intense area of investigation in medicinal chemistry [5,6,7]. Moreover, pharmaceutical research, manufacture, and regulation are enhancing the development of solid active ingredients, delivered as powders or tablets; however, many solid drugs which perform well in in vitro evaluation remain too insoluble for the body to absorb [8,9]. Most of the bioactive agents sold for pharmaceutical or food industries are salts [10,11], and in this context, ionic liquids (ILs) represent a promising class of drug candidates whose physicochemical and pharmaceutical properties can be easily tuned [12,13,14,15,16,17]. In this regard, the imidazolium skeleton can be transformed into ionic liquids with promisingly potent pharmacological properties [18,19,20,21]. Consequently, monoimidazolium [22,23,24] and bisimidazolium [25,26,27,28] salts have been explored as a new generation of antibacterial agents. In this context, amino acid-based monoimidazolium salts with good bacterial toxicity have been reported in the literature [29].

Our research group has an ongoing interest in the biomimetic and bioactive capacity of imidazole or imidazolium amino acid derivatives [30,31,32,33]. Herein, imidazole, monotopic, and ditopic imidazolium salts derived from *L*-valine and *L*-phenylalanine with different alkyl lengths in the amide substitution were synthesized and characterized comprehensively. The antibacterial activity of these amino acid-based imidazolium salts against Gram-negative *Escherichia coli* DH5-α (herein *E. coli*) and Gram-positive *Bacillus subtilis* 1904-E (herein *B. subtilis*) were evaluated and their cytotoxicity was also studied using a human embryonic kidney cell line (HEK-293). Finally, due to the amphiphilic character of these compounds and the strong tendency towards self-aggregation of ionic liquid-related surfactants based on imidazolium salts [34,35,36,37], we investigated the spontaneous aggregation behavior of these compounds in water and in bacterial cell culture medium. Through optical and scanning electron microscopy, as well as UV-vis and fluorescence spectroscopy, we have extracted structure–property relationships between the degree of aggregation/self-assembly of the *L*-valine and *L*-phenylalanine derivatives and their corresponding antibacterial activity and cytotoxicity [38].

## 2. Results

### 2.1. Synthesis

**Scheme 1:**
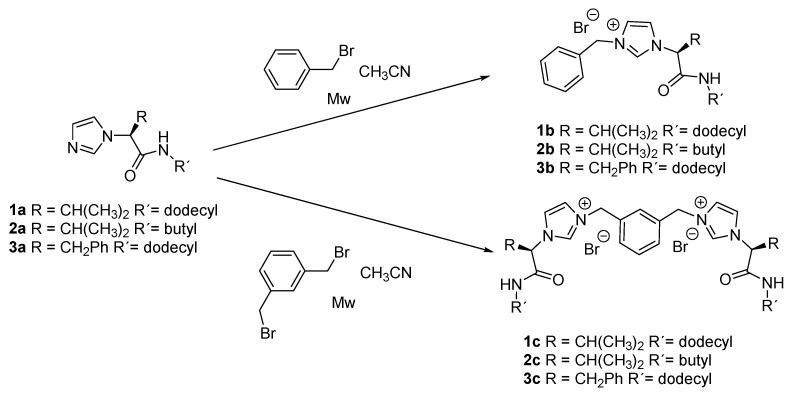
Synthesis of the amino acid-based imidazolium salts in this report.

The imidazole-amino acid derivatives **1a**, **2a**, and **3a** were obtained from the corresponding α-amino amide as previously described [31]. Monotopic and ditopic -imidazolium salts **1b–3b** and **1c–3c** were obtained in high yield by treatment of the corresponding imidazole with benzyl bromide or 1,3-bromomethylbenzene, respectively, as described in our previous publications (Scheme 1) [30,32].

### 2.2. Antibacterial and Cytotoxicity Studies

The in vitro antibacterial activities of the synthesized compounds were examined against *E. coli* and *B. subtilis*. Bacteria were incubated in culture media with varying concentrations of the examined compound and the antibacterial properties were determined by observation of the optical density at 560 nm (bacteriostatic activity) and by the Resazurin cell viability assay (bactericidal activity). The corresponding minimal bactericidal concentration (MBC) and minimum inhibitory concentration (MIC) values that were obtained are summarized in Table 1 (please refer to Table S1 for MIC and MBC values in μM).

The outer membrane of Gram-negative bacteria such as *E. coli* includes porins, which allow the passage of small hydrophilic molecules across the membrane, and lipopolysaccharide molecules that extend into extracellular space. Thus, the observed trend in the activity results could be explained by the relative lipophilicity of the compounds combined with their capacity to disrupt the cell membrane [39,40]. The relative lipophilicity of the compounds was determined theoretically using VCCLab and Molinspiration softwares (LogP values Table 1) and experimentally (retention time values from HPLC, Table 1, Figures S3–S8). The HPLC method used was first validated using different lipophilic commercial compounds with LogP values from 1 to 4.5 (Figure S3a). The structure–activity relationships of the compounds will be further discussed in Section 3.

**Figure 1:**
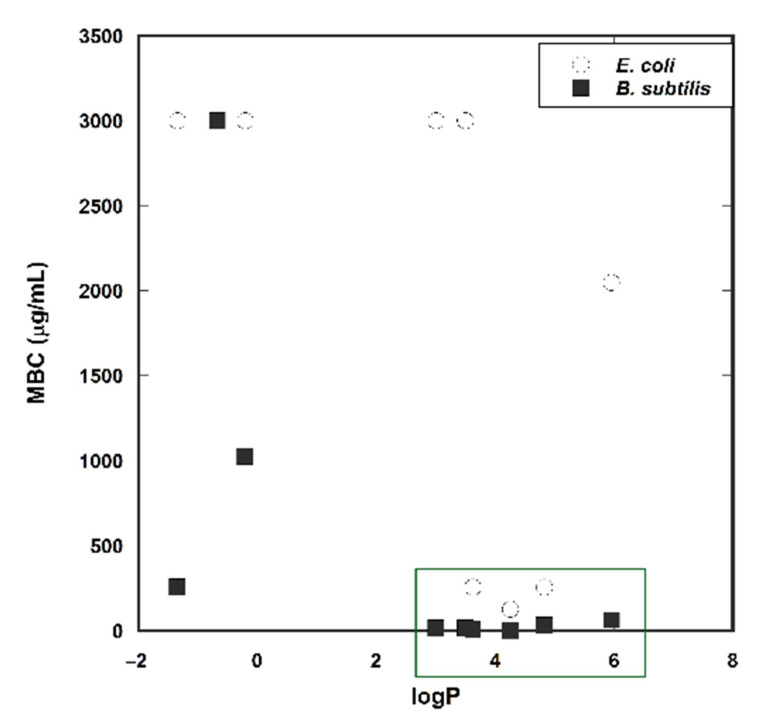
Plot of Log P vs. MBC. * For the sake of clarity, MBC values >2000 μg/mL are given the value of 3000 μg/mL.

The MBC values obtained were plotted against the logP values of the corresponding compounds to study the possible correlation between activity and lipophilicity (Figure 1).

**Figure 2:**
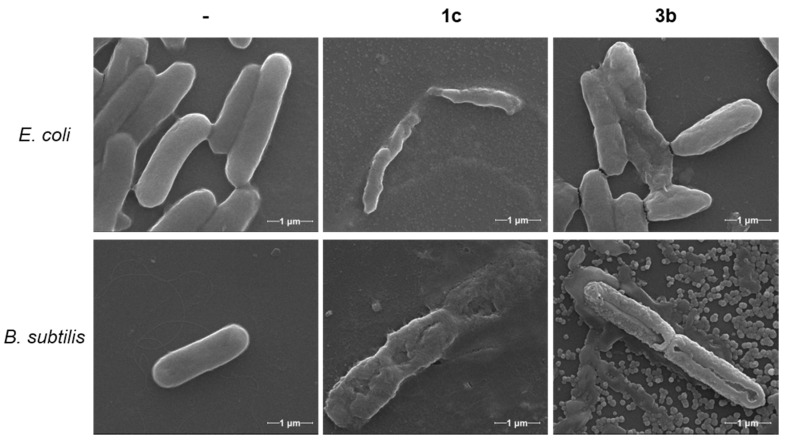
Scanning electron microscopy (SEM) images of *E. coli* and *B. subtilis* without treatment (-) and after incubation with compounds **1c** and **3b** at their corresponding MIC (60,000×). See Supporting Information for additional SEM images.

Electron microscopy is a powerful tool to further assess the effect of the imidazole derivatives and imidazolium salts on bacterial cell growth, inhibition, and death. Two compounds which showed from moderate to good antibacterial activity—the bisimidazolium salt **1c** and the monoimidazolium salt **3b**, respectively—were chosen for electron microscopy characterization. For these studies, *E. coli* and *B. subtilis* were inoculated for 20 h with each compound at their corresponding MIC (Figure 2) and ½ MIC (Figures S1 and S2) concentrations and fixed with glutaraldehyde.

### 2.3. Aggregation Studies

The self-assembly of the compounds in aqueous medium and in the bacterial cell culture medium (LB broth) was investigated by optical and scanning electron microscopy, as well as UV-vis and fluorescence spectroscopy.

#### 2.3.1. Fluorescence Spectroscopy

To investigate the microenvironment of the critical aggregation concentration (CAC) for self-assembly in water and in the bacterial cell culture medium by fluorescence, the intensity ratio of two of the peaks (*I*_1_*/I*_3_) of the pyrene fluorescence spectrum was used [43,44,45]. Plots of the pyrene *I*_1_*/I*_3_ ratio as a function of the total surfactant concentration show a typical sigmoidal decrease in the region where self-assembly takes place. At low concentrations, this ratio is larger as it corresponds to a polar environment for pyrene. When the surfactant concentration increases this ratio decreases rapidly, as the self-assembly favors the location of pyrene in a more hydrophobic environment, until reaching a roughly constant value because of the full incorporation of the probe into the hydrophobic region of the aggregates. Different approaches have been used to estimate CAC values from *I*_1_*/I*_3_ ratios [46]. The most common approach is the use of the break points, either directly or by extrapolating the values from the intersection of the two straight lines defined at the constant and variable regions of the *I*_1_*/I*_3_ sigmoidal curve [43,46,47,48]. As CAC represents the threshold of concentration at which self-aggregation starts, the corresponding value can be estimated from the break point at lower concentration (see Figures S9–S14) [49,50,51,52].

Fluorescence studies were carried out using MilliQ^®^ water and 1/1 MilliQ^®^ water/bacterial cell culture medium, because with the pure cell culture medium, a strong broad fluorescence emission band was observed precluding an accurate analysis. Furthermore, compound **3c** could not be studied due to solubility problems. The corresponding CACs obtained in water and in the 1/1 mixture of water/bacterial cell culture medium by fluorescence are shown in Table 2.

**Figure 3:**
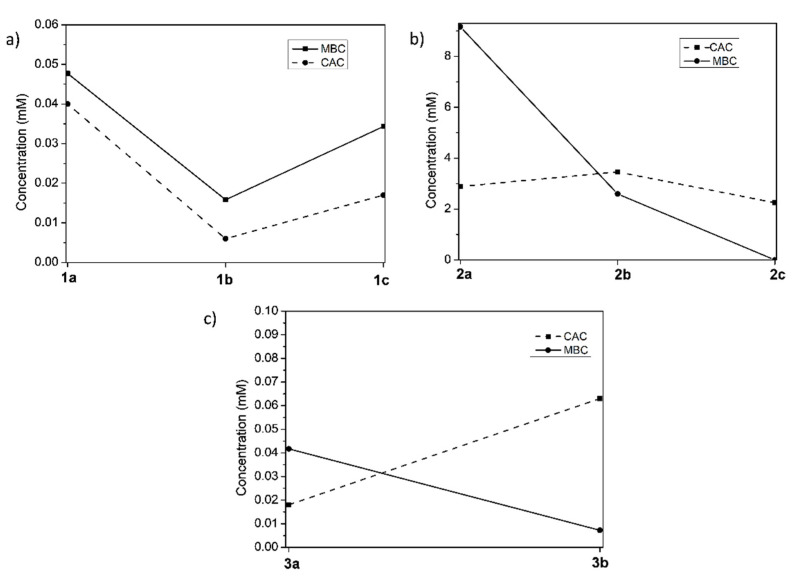
Correlation of MBC for *B. subtilis* and CAC for the different series of compounds in the bacterial cell culture medium: (**a**) valine derivatives with long alkyl chain; (**b**) valine derivatives with short chain; and (**c**) phenylalanine derivatives with long alkyl chain.

Furthermore, the MBC values of the compounds against *B. subtilis* were compared to their CAC (Figure 3). This comparison can shed light on the active form of the molecules exerting the antibacterial action, i.e., monomeric or aggregated structures.

#### 2.3.2. Optical Microscopy and Scanning Electron Microscopy (SEM)

**Figure 4:**
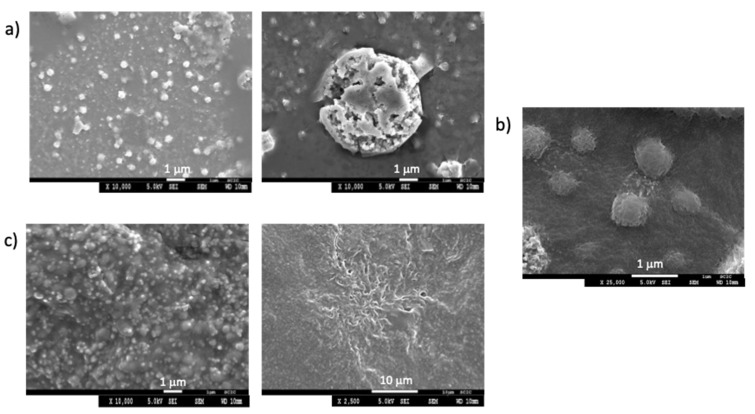
SEM images for **3b** (**a**) 0.5 mM in water; (**b**) 0.7 mM in 1/1 water/bacterial cell culture medium; and (**c**) 0.7 mM in the bacterial cell culture medium.

The morphology of the aggregates in water, in 1/1 water/bacterial cell culture medium, and in the cell culture medium at concentrations above the CAC, were studied by optical microscopy (Figures S15–S17) and SEM (Figure 4 and Figure S18).

#### 2.3.3. UV-Vis Spectroscopy

**Figure 5:**
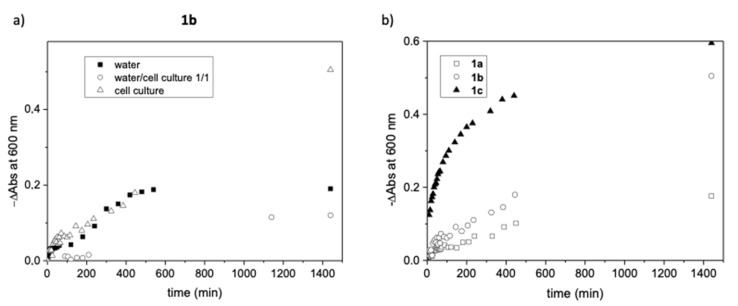
(**a**) Change in absorbance at 600 nm with respect to time for **1b** at 1 mM in different media. (**b**) Change in absorbance at 600 nm with respect to time for **1a–1c** at 1 mM in the bacterial cell culture medium.

The aggregation and stability of the aggregates in the different solvents, water, 1/1 water/bacterial cell culture, and bacterial cell culture medium for **1a-c** were studied by UV-vis at 25 °C measuring the absorbance at 600 nm, for 1 mM colloidal solutions (Figure 5, Figures S19 and S20) [53,54].

## 3. Discussion

The Gram-positive cell wall is composed of a thick, multilayered peptidoglycan sheath outside of the cytoplasmic membrane, while the Gram-negative cell wall is composed of an outer membrane linked by lipoproteins to thin, mainly single-layered peptidoglycan. The peptidoglycan is located within the periplasmic space that is created between the outer and inner membranes [55]. It therefore follows that all the tested compounds were more active against *B. subtilis* (Gram-positive) than *E. coli* (Gram-negative) (i.e., see Table 1, entries 1 and 7). Compound **3b** (entry 8) proved to be the most active antibacterial agent possessing an MBC as low as 4 and 128 μg/mL against *B. subtilis* and *E. coli* respectively; while those compounds with shorter alkyl chains presented the lowest activity. Some of the compounds (Table 1, entries 2, 3, 6, 7, and 8) presented MIC values lower than MBC values. An antimicrobial compound is considered to be bactericidal whenever its MBC to MIC ratio is less than or equal to four. Compounds with MBC/MIC >4 are considered to be bacteriostatic [56]. In all the cases in which we could obtain exact MBC and MIC values (Table 1, entries 2, 3, 8 and 9), their ratio was equal to or minor than four. Therefore, compounds **1b**, **1c**, **3b**, and **3c** can be considered to possess true bactericidal activity.

The structural element which clearly relates to a better activity is the use of longer alkyl chains (compounds **1** and **3**) in contrast to shorter alkyl chains (compounds **2**), probably due to an increased lipophilicity. The toxicity towards miscellaneous bacterial strains of alkyl imidazolium salts has been reported to increase with the length of the alkyl chain [57]. The monoimidazolium salts with these longer alkyl chains (**1a-b** and **3a-b**) were more active against *B. subtilis* and *E. coli* than the bisimidazolium counterparts, with the monotopic salt **3b** showing the lowest MIC and MBC values (entries 2 and 8, Table 1), this indicates that the introduction of two hydrophobic alkyl chains contributes greatly to decrease the activity, opposite to the trends observed in the literature [27].

Regarding amino acid nature, the phenylalanine monotopic salt with long alkyl chain (**3b**) presented lower MIC and MBC values against *E. coli* than the analogous valine compound (**1b**) (entries 2 and 8, Table 1), however for ditopic salts, the behavior is the opposite, **3c** presented higher MIC and MBC values than **1c** (entries 3 and 9, Table 1).

Although a similar biological activity for the Gram-positive and Gram-negative organisms is preferred, it is not always the case with different strains of microorganisms. Some authors have described that Gram-positive organisms preferred a more lipophilic molecule than the Gram-negative ones [58]. This has been attributed to the difference in the cell outer membrane between bacterial types and strains: while Gram-positive bacteria have a very simple cell wall, the outer membrane of Gram-negative bacteria contains lipopolysaccharides which are cross-bridged by divalent cations, adding strength to the membrane and impermeability to lipophilic molecules. This agreed with the results obtained in Table 1 where the more lipophilic compounds showed less activity against *E. coli*.

A good correlation was obtained between the theoretical logP, calculated using the average values from VCCLab software and Molinspiration, and the retention time observed from HPLC (Figure S1). The activity observed against *B. subtilis* increases for compounds with logP > 3 with MBC ≤64 mg/mL. However, MBC values greater than 2000 μg/mL were obtained for both lipophilic and lipophobic compounds, with the exception of the lipophilic compounds **1b**, **1c**, and **3b** which present lower MBC values (Figure 1).

From the SEM images in Figure 2, both bacteria strains incubated with compounds **1c** and **3b** show clear signs of damage: from morphological changes to disruption of the cell membrane and leakage of cytoplasmatic material, ending with the disintegration of the bacteria into small fragments. Most images, especially of *B. subtilis*, show an “implosion” of bacteria, with a marked depression in the middle of the cell (also refer to Figures S1 and S2). In some of the images, aggregates of the compounds can be seen surrounding the bacteria, many of which are attached to the cell membrane.

Human embryonic kidney cells, HEK-293, were chosen as a cell model to evaluate the cytotoxicity of the compounds. The HEK-293 cells were incubated in the cell culture medium with varying concentrations of the examined compound and the impact of treatment was measured using the MTT cell viability assay [46]. The results indicated that compounds **1a–1c**, **3a**, and **3c** were considerably toxic with IC_50_ values lower than 36 μM (Table 1 and Table S1). Surprisingly, compound **3b** derived from phenylalanine was less toxic to the HEK-293 cell line at concentrations 15 times higher than the MIC and MBC for the *B. subtilis* strain (Table 1, entry 8) [59].

Comparing results with the commercial antibiotic alamethicin, the corresponding MIC value of **3b** against *B. subtilis* is lower than alamethicin, (Table 1, entry 10) [41], whereas the toxicity of alamethicin against MRC-5 human cells is similar to the cytotoxicity of the imidazolium salt **3b** against HEK-293 line [42].

To gain a more detailed understanding on the mechanism of cytotoxicity of these imidazole and imidazolium salts on bacteria, we studied the possible structure–activity relationship between their antibacterial activities and their aggregation behavior in bacterial cell medium [23,38].

From pyrene fluorescence studies in pure water, the plot of the *I*_1_*/I*_3_ ratio for the corresponding emission spectra vs. concentrations showed one single break point (Figure S9, Figure S11, and Figure S13) reaching in all cases values of *I*_1_*/I*_3_ ≈ 1.3 or lower after the break point. However, in the bacterial cell culture medium, the plot of the *I*_1_*/I*_3_ ratios for the corresponding emission spectra vs. concentration presented two single break point and, in some cases, even three points. The two break points observed for all the compounds suggest the presence of two different processes. The first one takes place at *I*_1_*/I*_3_ values observed for pyrene in the absence of compounds (*I*_1_*/I*_3_ = 1.16 in water/1/1 bacterial cell culture medium) and reveals that pyrene is fully exposed to the polar solvent mixture in this first aggregation step. At the second break point, at higher concentrations, this ratio reaches lower values suggesting the formation in this region of aggregates in which the probe molecule is less solvent exposed [60]. For example, for compound **1b**, the first break point at 6 μM leads to aggregates with an appreciable solvent exposed probe (*I*_1_*/I*_3_ ≈ 1.1) and the second process starts at *ca.* 48 μM affording aggregates, providing a low polarity microenvironment to pyrene, reaching *I*_1_*/I*_3_ values ≈ 0.8 for 1 mM concentration.

The CAC values obtained for compounds with long alkyl chain were in the μM range while for compounds **2a-c** with short alkyl chains, the CAC values obtained were in the mM range (i.e., entries 1 and 4, Table 2), where the lowest CAC values for the ditopic salts were found in water. In general, for the imidazole and monotopic salts, changing the medium from water to water/bacterial cell culture medium led to a decrease in the CAC values (i.e., Table 2, entries 1 and 2), however for ditopic salts, the CAC values did change significantly (Table 2, entries 3 and 6).

Comparing the first CAC values obtained in 1/1 water/bacterial cell culture medium and the MBC for *B. subtilis* for the different series of compounds in Figure 3, it can be observed as compounds **1a–1c** presented the CAC below the MBC, implying that these compounds exist in an aggregated form at the MBC concentration, meaning fewer imidazolium monomers will be present at these concentrations, less than is needed to produce a significant biologic effect, thus increased overall concentrations are needed to obtain the desired bactericidal effects if the monomeric form is the responsibility of the corresponding bioactivity. However, different behavior was observed for the series **3a–3b** derived from phenylalanine. Figure 3c shows how the CAC line intersects the MBC line, indicating that compound **3b** is not aggregated at the corresponding MBC value and exerts a high bactericidal effect, as observed in Table 2 (entry 8). Finally, for the **2a–2c** series, the CAC is below the MBC for **2a** but above for **2b** and **2c**, illustrating that the monomeric form is responsible for the corresponding antibacterial activity (Table 2, entry 6).

In addition to the results above, the compounds containing dodecyl chains can easily align with lipids and hence, accumulate within the bacterial cell membrane. In this regard, compounds with longer alkyl tails have CACs in the μM range in the bacterial cell culture medium and thus easily self-assemble, leading to an easy accumulation within the cell membrane. Therefore, resulting in a lower effective concentration at the site of action within the cellular cytoplasm lower. This accumulation could lead to a biocidal mechanism based on [38], as is observed in Figure 2. Furthermore, it appears that the shorter chain length results in reduced membrane interaction and an energetically unfavorable micelle formation, meaning low self-assembling capability, which leads to a lower overall bacterial cytotoxicity as seen from the corresponding MIC and MBC values (Table 1).

Consequently, by comparing the MBC for *B. subtilis* and CAC of the imidazole and imidazolium series, this study has provided a better understanding of the relationship between the biological activities of these compounds correlated with their aggregation capabilities. The results demonstrate that the compounds with longer alkyl chains provide excellent antimicrobial activity although most of them are aggregated at the antimicrobial response concentration, with only compound **3b** existing in its monomeric form at its corresponding MBC value.

Optical microscopy confirmed the formation of spherical aggregates between 0.5–20 μm diameter in size in the three different media (Figures S15–S17). Regarding the medium, in general, in the culture medium, the dispersity of the aggregates decreased for compounds with longer alkyl chains (see Figures S15 and S17). Furthermore, in the culture medium, compounds **2a-c** and **3b** were able to form worm-like aggregates at the studied concentrations (Figures S16 and S17).

SEM images for **1c** and **3b** in water, in 1/1 water/bacterial cell culture medium and in the bacterial cell culture medium revealed the formation of different aggregates morphologies. Compound **1c** produced spherical aggregates with <3 μm diameter size in all three media, with the aggregates in water being more distorted (Figure S18). Compound **3b** was able to form spherical aggregates in water and water/bacterial cell culture medium (Figure 4a,b), while in the pure culture medium, different morphologies were observed, such as spherical aggregates <1 μm in diameter coexisting with fibrillary aggregates (Figure 4c). When viewed at higher magnification, it is observed that the larger spherical aggregates consisted of several smaller aggregates or dendritic fibrillary aggregates for **3b** and **1c,** respectively (Figure 4 and Figure S18).

Regarding the stability of the aggregates formed, studies by UV-vis spectroscopy for **1a-c** are gathered in Figure 5. Figure 5a shows the change in absorbance (−∆A_600_) of compound **1b** (1 mM) at 600 nm with respect to time in the different media. The initial rate for the change in absorbance associated to the destabilization of the aggregates is defined by the slope of the linear region of the initial −∆A_600_ versus time plot. The slope and the total change in the absorbance was the smallest when using the water/bacterial cell culture medium, being the rate at the initial region for pure water and bacterial cell culture medium in the same ranges. However, it must be highlighted that the absorbance decreases until reaching a zero value after 500 min for pure water. A different behavior was obtained for **1a** and **1c** (see Figures S19 and S20). For compound **1a**, the absorbance at 600 nm decreased with time in the three media with similar rates, while for **1c**, the rate followed the order water > bacterial cell culture medium > 1/1 water/bacterial cell culture medium, reaching almost zero values in the tree media after 24 h.

Overall, the results obtained show that in the pure culture medium, the stability of the aggregates follows the order **1a** > **1b** > **1c** (Figure 5b), indicating that the introduction of the two headgroups and hydrophobic alkyl chains in **1c** contributes to a minor stabilization of the aggregates in this medium.

## 4. Materials and Methods

### 4.1. Materials

#### 4.1.1. Reagents and Culture Media

Resazurin sodium salt and dimethyl sulfoxide (DMSO) were bought from Sigma-Aldrich. Luria-Bertani (LB) liquid broth (Miller’s formulation) and nutrient broth (NB) were freshly prepared and sterilized by autoclave. Broth powders were bought from Scharlab. Tryptone soy agar plates were purchased from Thermo Scientific. Glutaraldehyde was purchased in solution at 25% in H_2_O and Grade II from Sigma Aldrich and used as provided. Phosphate buffer was prepared from the solid salts NaH_2_PO_4_ and Na_2_HPO_4_, both purchased from Aldrich at qualities 99% and 99.5% respectively, by dissolving them in MilliQ^®^ water and adjusting pH with NaOH and HCl solutions.

Cell culture media for cytotoxicity studies were purchased from Gibco (Grand Island, NY, USA). Fetal bovine serum (FBS) was obtained from HyClone (UT, USA). Supplements and other chemicals not listed in this section were obtained from Sigma Chemical Co. (St. Louis, MO, USA). Plastics for cell culture were supplied by Thermo Scientific BioLite (Madrid, Spain). All tested compounds were dissolved in DMSO at a concentration of 10 mM and stored at −20 °C until use. HEK-293 cell lines were maintained in Dulbecco’s modified Eagle’s medium (DMEM) containing glucose (1 g/L), glutamine (2 mM), penicillin (50 μg/mL), streptomycin (50 μg/mL), and amphotericin B (1.25 μg/mL) supplemented with 10% FBS.

Reagents and solvents, including NMR solvents, were purchased from commercial suppliers and were used without further purification except for pyrene, used for fluorescence studies, that was crystallized twice from methanol. Deionized water was obtained from a MilliQ^®^ equipment (Burlington, MA, USA). Imidazoles **1a** and **2a** and imidazolium salts **1b** and **1c** were prepared as previously described [30,32].

#### 4.1.2. Synthesis and Characterization

Imidazole **3a** and compounds **2b–c** and **3b–c** were prepared following the synthetic protocols.

*General procedure for compound **3a**:* To a mixture of glyoxal (40% aq., 1.1 equiv, 2.6 mL) and formaldehyde (37% aq., 5.0 equiv., 7.7 mL), the (*S*)-2- amino-N-dodecyl-3- phenylpropanamide compound (1.0 equiv., 6.9 g, 20.8 mmol) and ammonium acetate (1.1 equiv, 1.8 g, 23.4 mmol) were dissolved previously in methanol and added. The reaction mixture was stirred at room temperature for 48 h. The solvent was evaporated under reduced pressure and the resulting crude residue was treated with saturated Na_2_CO_3_ solution, extracted with CH_2_Cl_2_ (3×), dried with anhydrous MgSO_4_, filtered, and concentrated.

*General procedure for compounds **2b–3b**:* To a mixture of compound **2a–3a** (1.1 equiv) and bromomethylbencene (1.0 equiv) were dissolved in acetonitrile (5 mL). The reaction was carried out under microwave irradiation using 120 W, 1.72 × 10^6^ Pa, 150 °C, and 1 h. After solvent evaporation, the remaining solid was washed with diethyl ether (×3) to afford the desired compound.

*General procedure for compounds **2c–3c**:* To a mixture of compound **2a–3a** (2.2 equiv) and 1, 3-(bis-bromomethyl)benzene (1.0 equiv) were dissolved in acetonitrile (5 mL). The reaction was carried out under microwave irradiation using 120 W, 1.72 × 10^6^ Pa, 150 °C, and 1 h. After solvent evaporation, the remaining solid was washed with diethyl ether (×3) to afford the desired compound.

*Compound **3a**:* yellow liquid (7 g, 88%), [α]^25^_D_ = −7.11 (c = 0.01, MeOH); m.p = 56.1 °C. ^1^H NMR (400 MHz, CDCl_3_ and CD_3_OD) δ 7.26–7.07 (m, 4H), 7.00 (m, 1H), 6.97–6.89 (m, 3H), 6.48 (t, J = 5.7 Hz, NH), 4.67 (dd, J = 9.1, 6.0 Hz, 1H), 3.46 (dd, J = 14.0, 6.0 Hz, 1H), 3.19–3.00 (m, 3H), 1.30 (m, 2H), 1.26–1.04 (m, 18H), 0.85–0.77 (m, 3H). ^13^C NMR (101 MHz, CDCl_3_) δ 168.3, 134.0, 136.3, 129.6, 128.8, 128.7, 127.2, 118.0, 62.9, 39.8, 39.3, 31.9, 29.6, 29.6, 29.5, 29.3, 29.2, 26.8, 22.7, 14.1. MS (ESI) (m/z) calcd. for C_24_H_37_N_3_O [M+H]^+^ = 384.3; found 383.4 (100%), 767.7 (35%, [M+M+H]^+^). IR (ATR) = 3309, 2953, 2919, 2850, 1656, 1549, 1493, 1469, 1454 cm^−1^. Calculated for C_24_H_37_N_3_O·4H_2_O: C 63.27, H 9.96, N 9.22; found C 62.97, H 9.74, N 9.58.

*Compound **2b**:* yellow oil (150 mg, 93%); [α]^25^_D_ = 41.07 (c = 0.01, MeOH); m.p. 33 °C. ^1^H NMR (400 MHz, CDCl_3_) δ 9.93 (s, 1H), 8.62 (t, J = 5.7 Hz, 1H), 7.75 (t, J = 1.8 Hz, 1H), 7.38–7.21 (m, 5H), 7.11 (s, 1H), 5.66 (d, J = 10.6 Hz, 1H), 5.47–5.27 (dd, J = 10.6, 2.2 Hz, 2H), 3.34–3.17 (m, 1H), 3.12–2.96 (m, 1H), 2.45–2.37 (m, 1H), 1.54–1.40 (m, 2H), 1.32–1.21 (m, 2H), 1.03 (d, J = 6.5 Hz, 3H), 0.81 (t, J = 7.3 Hz, 3H), 0.76 (d, J = 6.6 Hz, 3H). ^13^C NMR (101 MHz, CDCl_3_) δ 167.0, 136.1, 131.8, 130.1, 129.8, 128.6, 121.8, 120.9, 67.7, 53.9, 39.6, 31.2, 31.0, 20.2, 18.8, 18.3, 13.7. MS (ESI) (m/z) calcd. for C_19_H_28_N_3_O [M]^+^ = 314.2; found 314.5 (100%); IR (ATR)= 3220, 3063, 2962, 2933, 2873, 1672, 1550, 1497, 1456, 1327, 1225, 1152 cm^−1^. Calculated for C_19_H_28_N_3_OBr: C 57.87, H 7.16, N 10.66; found C 57.80, H 6.98, N 11.01.

*Compound **3b**:* yellow viscous solid (129 mg, 90%); [α]^25^_D_ = −31.07 (c = 0.01, MeOH); m.p= 16 °C. ^1^H NMR (300 MHz, CDCl_3_) δ 9.53 (s, 1H), 8.59 (t, J = 5.5 Hz, NH), 7.74 (s, 1H), 7.41–7.14 (m, 7H), 7.04–6.95 (m, 2H), 6.92 (s, 1H), 6.55 (m, 1H), 5.14 (q, J = 14.7 Hz, 2H), 3.51–2.95 (m, 4H), 1.83 (s, 3H), 1.44 (m, 2H), 1.17 (s, 16H), 0.92–0.70 (m, 3H). ^13^C NMR (101 MHz, CDCl_3_) δ 166.6, 136.1, 134.3, 131.9, 129.8, 129.7, 129.1, 129.0, 128.2, 127.5, 121.8, 120.9, 62.3, 53.6, 40.0, 39.0, 31.9, 29.7, 29.6, 29.5, 29.4, 29.2, 28.9, 27.0, 22.7, 14.1. MS (ESI) (m/z) calcd. for C_31_H_44_N_3_O [M]^+^ = 474.4; found 474.7 (100%); IR (ATR)= 3297, 3061, 2966, 2922, 2851, 1654, 1557, 1495, 1453 cm^−1^. Calculated for C_31_H_44_N_3_OBr·H_2_O: C 65.02, H 8.10, N 7.34; found C 65.46, H 8.24, N 7.68.

*Compound **2c**:* yellow oil (104 mg, 90%); [α]^25^_D_ = 6.93 (c = 0.01, MeOH); m.p.= 85 °C. ^1^H NMR (500 MHz, CDCl_3_) δ 10.01 (s, 2H), 8.40 (t, J = 5.6 Hz, 2H), 8.12 (s, 2H), 7.68 (d, J = 1.3 Hz, 2H), 7.47–7.36 (m, 2H), 7.34–7.23 (m, 2H), 5.73 (d, J = 14.4 Hz, 2H), 5.55–5.38 (dd, J = 10.7 Hz, J = 3.5 Hz, 4H), 3.37–3.25 (m, 2H), 3.15–3.00 (m, 2H), 2.53–2.38 (m, 2H), 1.64–1.43 (m, 4H), 1.39–1.20 (m, 4H), 1.08 (d, J = 6.6 Hz, 6H), 0.87 (t, J = 7.3 Hz, 6H), 0.82 (d, J = 6.7 Hz, 6H). 13C NMR (126 MHz, CDCl_3_) δ 206.8, 166.7, 136.5, 134.1, 130.6, 130.5, 129.9, 122.4, 120.8, 77.3, 77.0, 76.8, 68.1, 53.2, 39.5, 31.1, 30.9, 30.9, 20.1, 18.8, 18.4, 13.6. MS (ESI) (m/z) calcd. for C_32_H_50_N_6_O_2_ [M]^2+^ = 275.2; found 275.3 (100%); IR (ATR)= 3412, 3228, 3125, 3066, 2962, 2933, 2873, 1671, 1550, 1465, 1360, 1298, 1226, 1152 cm^−1^. Calculated for C_32_H_50_N_6_O_2_Br_2_·2H_2_O: C 51.48, H 7.29, N 11.26; found C 50.84, H 7.32, N 11.46.

*Compound **3c**:* yellow viscous solid (126 mg, 82%); [α]^25^_D_ = 17.33 (c = 0.01, MeOH); m.p.= 60 °C. ^1^H NMR (300 MHz, CDCl_3_) δ 9.47 (s, 2H), 8.21 (t, J = 5.7 Hz, NH), 7.81–7.51 (m, 6H), 7.29–7.06 (m, 16H), 6.13 (t, J = 8.0 Hz, 2H), 5.31 (s, 4H), 3.37 (dd, J = 13.6, 7.2 Hz, 2H), 3.25–3.10 (m, 4H), 3.00–2.84 (m, 2H), 1.41–1.29 (m, 4H), 1.23–1.05 (m, 32H), 0.80 (m, 6H). ^13^C NMR (101 MHz, CDCl_3_) δ 166.3, 136.3, 134.3, 133.6, 130.6, 130.3, 129.7, 129.2, 128.9, 127.5, 122.3, 121.0, 62.6, 53.0, 39.9, 38.9, 31.9, 29.7, 29.7, 29.6, 29.5, 29.4, 29.2, 28.9, 26.9, 22.7, 14.1. MS (ESI) (m/z) calcd. for C_56_H_82_N_6_O_2_ [M]^2+^ = 435.3; found 435.7 (100%); IR (ATR)= 3294, 3063, 2923, 2852, 1656, 1554, 1495, 1454, 1362 cm^−1^. Calculated for C_56_H_82_N_6_O_2_Br_2_: C 65.23, H 8.02, N 8.15; found C 65.76, H 8.57, N 8.34.

#### 4.1.3. Microorganisms and Growth Conditions

Two bacterial strains were used in the antibacterial assays: *Escherichia coli* DH5α as a Gram-negative model and *Bacillus subtilis* 1904-E as a Gram-positive model. Both bacterial strains were donated to our laboratory and can be provided on request by contacting the corresponding authors. Both bacterial strains were incubated at 37 °C and the pre-inoculum incubation time was of 24 h. Liquid Luria-Bertani (LB) medium was used for *E. coli* DH5α and nutrient broth (NB) for *B. subtilis*.

### 4.2. Methods

#### 4.2.1. Bacterial Proliferation Assay in Presence of Imidazole Derivatives

The bacteria cell bank suspensions were thawed and inoculated in the appropriate liquid broth for 24 h at 37 °C with mild agitation. A dilution from these culture solutions was used for the following tests, corresponding to an inoculum of 1 × 10^7^ CFU/mL. Stock solutions of all the tested compounds were prepared in DMSO at a concentration of 100 mg/mL, aliquoted, and stored at −20 °C.

*(A) Bacterial growth inhibition assay:* Conditions here described are for testing 6 different concentrations of the compounds, with triplicates of each condition. Therefore, 4 compounds were tested per plate. An adapted version of the microdilution method was used. Firstly, the imidazole derivatives were dissolved in the corresponding broth at 2× the highest tested concentration. Then, 100 μL of the 2× solutions were added to the first (A) and second (B) row wells of a 96-well plate. In addition, 100 μL of liquid medium had been previously added to rows B to F. Subsequent dilutions at 1:2 are prepared in rows B to F, by withdrawal of 100 μL from the previous row (more concentrated) to the next row (half diluted), mixing well. Then, 100 μL were discarded from the last row (F). By now, there are 100 μL in each well, and 100 μL of bacterial suspension at 10^7^ CFU/mL were added to each well. Then, the 96-well plates were incubated for 24 h at 37 °C under mild agitation. Bacterial growth was controlled both by visual observation of the turbidity in each well and by measuring the optical density (OD) at 560 nm at time 0 h and 24 h. Results are recorded as the lowest concentration of antimicrobial agent that inhibits visible growth of the bacteria, and were compared with the OD variation of a control culture containing *E. coli* or *B. subtilis* (+ control) and of solution of the tested compounds without bacteria (- control).

*(B) Bacterial cell viability assay:* Cell viability was analyzed using a Resazurin (7-Hydroxy-3H-phenoxazin-3-one 10-oxide) assay in a 96-well plate. Once the bacterial cultures of growth inhibition assay had been grown for a total of 24 h, 25 μL of a 0.1 mg/mL resazurin (prepared in LB or NB medium) were added to each well and incubated in the dark at 37 °C for 1 h under stirring. Resazurin has a blue color at the testing pH and turns pink when reduced by the viable bacteria to resorufin. Therefore, pink wells indicate metabolizing bacteria, while blue wells are indicative of bacteria that have lost their ability to convert resazurin to resorufin. Different controls were made in order to corroborate the MBC value obtained by the resazurin assay. The change of color was confirmed at 1, 4, and 24 h after its addition. The viability of bacteria was verified (either confirmed or rejected) by the colony plate-counting method, by seeding 10 μL from the cell culture onto tryptone soy agar plates and observing the presence or absence of bacterial growth after 24 h at 37 °C.

#### 4.2.2. Log P Calculation and Retention Time Determination

LogP values for the different compounds were calculated using VCCLab (ALOGPS 2.1) and Molinspiration (miLogP2.2) softwares. We used LogP as the average of these values. The protonated forms for the imidazole derivatives were considered. Reverse phase HPLC (equipment: Agilent technologies 1100 series, column: Xterra MS C18 4.6 × 150 mm (5 μmol/L)) was also used for measuring the relative lipophilicity of these compounds, since the retention time of each molecule on the reverse phase column is related to its lipophilicity. All the products were dissolved in MeOH at 2 mmol/L concentration and eluted using 70/30 acetonitrile/water and 0.1% of formic acid for 15 min and flow rate 0.2 mL/min at 25 °C. λ used was 254, 280, and 220 nm taking the corresponding chromatogram with higher mAU (see Supporting Information).

#### 4.2.3. H NMR Studies

NMR experiments were carried out on a Varian INOVA 500 spectrometer (500 MHz for ^1^H and 125 MHz for ^13^C), on a Bruker Avance III HD 400 spectrometer (400 MHz for ^1^H and 100 MHz for ^13^C) or on a Bruker Avance III HD 300 spectrometer (300 MHz for ^1^H and 75 MHz for ^13^C) at 25 °C. Chemical shifts are reported in ppm using TMS as the reference.

#### 4.2.4. Fluorescence Spectroscopy Measurements

Pyrene was used as a fluorescence probe to determine the CAC of the compounds in water and 1/1 water/bacterial cell culture medium at 25 ± 1 °C. Fluorescence measurements were performed with a Spex Fluorolog 3-11 instrument equipped with a 450 W xenon lamp (right angle mode). Firstly, a stock pyrene solution of 1.98 × 10^−4^ mol/L was prepared in ultrapure methanol. Then, solutions of the imidazole and imidazolium salt compounds (ranging from 6 to 3 × 10^−3^ mmol/L) were prepared in different vials and 5 μL of pyrene solution was transferred into the vials, reaching a final pyrene concentration of 9.89 × 10^−7^ mol/L in each vial. Fluorescence spectra of pyrene were recorded from 200 to 650 nm after excitation at 337 nm, and the spectra were not corrected for the Xe lamp spectral response. The slit width was set at 5 nm for both excitation and emission. The peak intensities at 373 and 385 nm were determined as *I*_1_ and *I*_3_, respectively. The ratios of the peak intensities at 373 and 385 nm (*I*_1_/*I*_3_) for the emission spectra were recorded as a function of the logarithm of concentration. The CAC values were taken from the break point. Samples were excited with a 337 nm NanoLED.

#### 4.2.5. Optical Images

Images were recorded with OLYMPUS COVER-018 microscopy, BX51TF model, at 25 °C. Experiments were carried out in water, 1/1 water/bacterial cell culture medium, and in bacterial cell culture medium.

#### 4.2.6. Scanning Electron Microscopy (SEM)

SEM images of the compounds were obtained using a JEOL 7001F microscope with a digital camera; while SEM images of the incubated bacteria were obtained using an Inspect F50 microscope, at 10 kV and spot size of 3.0, with a digital camera. Bacteria solutions at ca. 0.5 × 10^7^ CFU/mL were incubated overnight without and with compounds **1c** and **3b** at their ½ MIC and MIC. After this, bacteria were washed with sterile PBS and fixed by incubation for 2 h in a 2.5% glutaraldehyde solution in phosphate buffer 10 mmol/L at pH 7.2. The fixed bacteria were subsequently washed once with phosphate buffer saline solution and four times with MilliQ water to remove any residual salts and glutaraldehyde. Finally, bacteria were resuspended in MilliQ water and 10 μL of these solutions were placed on silicon wafers and allowed to dry by evaporation overnight. Samples were coated with platinum using the sputtering technique in which microscopic particles of platinum are rejected from the surface after the material is itself bombarded by energetic particles of a plasma or gas. Experiments were carried out in water, 1/1 water/bacterial cell culture medium, and in bacterial cell culture medium.

#### 4.2.7. UV-Vis Spectroscopy

UV-Vis absorption spectra of the colloidal solutions were recorded on a Hewlett-Packard 8453 spectrophotometer at 25 °C. Experiments were carried out in water, 1/1 water/bacterial cell culture medium, and in bacterial cell culture medium.

#### 4.2.8. Cell Proliferation Assay for Cytotoxicity Studies

In 96-well plates, 3 × 10^3^ HEK-293 cells per well were seeded and incubated with serial dilutions of the tested compounds (from 200 to 0.2 μM) to a total volume of 100 μL of their growth media. The 3-(4,5-dimethylthiazol-2-yl)-2,5-diphenyltetrazolium bromide (MTT; Sigma Chemical Co.) dye reduction assay in 96-well microplates was used, as previously described [18]. After 2 days of incubation (37 °C, 5% CO_2_ in a humid atmosphere), 10 μL of MTT (5 mg/mL in phosphate-buffered saline, PBS) was added to each well, and the plate was incubated for a further 3 h (37 °C). The supernatant was discarded and replaced by 100 µL of DMSO to dissolve formazan crystals. The absorbance was then read at 540 nm by Multiskan^TM^ FC microplate reader. For all concentrations of compound, cell viability was expressed as the percentage of the ratio between the mean absorbance of treated cells and the mean absorbance of untreated cells. Three independent experiments were performed, and the IC_50_ values (i.e., concentration half inhibiting cell proliferation) were graphically determined using GraphPad Prism 4 software.

Statistical analysis: GraphPad Prism v4.0 software (GraphPad Software Inc., La Jolla, CA, USA) was used for statistical analysis. For all experiments, the obtained results of the triplicates were represented as means with standard deviation (SD).

## 5. Conclusions

A series of novel imidazole and imidazolium salts derived from *L*-valine and *L*-phenylalanine containing different hydrophobic groups have been synthesized and their antibacterial activity studied against *E. coli* and *B. subtilis*. The results demonstrate that an optimum lipophilicity of the alkyl chain and the amino acid side chain is needed to achieve antibacterial activity. The compounds presented better antibacterial activity against *B. subtilis* than *E. coli*, where compound **1a-1b** and **3a-3b** were the most active against *B. subtilis*, showing MBC values corresponding to 16 μg/mL or lower. Monotopic compound **3b** was 15 times less active against human embryonic kidney cells HEK-293 than toward *B. subtilis,* thus demonstrating its potential as an effective antibacterial agent with good biocompatibility. Aqueous aggregation studies revealed CAC values for compounds **1a–1c** and **3a–3c** in the μM range in water alone, however these CAC values decreased for imidazole and monotopic species when water was replaced with bacterial cell culture medium. Optical microscopy and SEM images confirmed the formation of these spherical aggregates. It is important to note that most of the bioactive compounds were aggregated to some extent at their MIC/MBC concentrations, however the monotopic compound **3b** was not aggregated at its corresponding MBC, suggesting that the monomeric species was responsible for the observed antibacterial activity.

## Figures and Tables

**Table 1:** Minimum inhibitory concentration (MIC, μg/mL), minimal bactericidal concentration (MBC, μg/mL), the half maximal inhibitory concentration IC_50_ (μg/mL) values and the partition coefficient log P values and HPLC retention times for the different compounds.

Entry	Compound	LogP ^a^	Retention Time (min) ^b^	*E. coli*		*B. subtilis*		HEK-293 IC_50_ μg/mL
				MIC μg/mL ^e^	MBC μg/mL ^e^	MIC μg/mL ^e^	MBC μg/mL ^e^	
1	**1a**	3.01 ^c^	6.1	>2000	>2000	16	16	3.2 ± 0.5
2	**1b**	3.63 ^d^	6.2	128	256	4	8	0.8 ± 0.2
3	**1c**	4.83 ^d^	7.7	256	256	16	32	18 ± 4
4	**2a**	−0.67 ^c^	3.4	>2000	>2000	>2000	>2000	>45
5	**2b**	−0.20 ^d^	3.7	>2000	>2000	>1000	>1000	>79
6	**2c**	−1.34 ^d^	3.1	1000	>2000	128	256	>142
7	**3a**	3.50 ^c^	6.7	1000	>2000	16	16	7.3 ± 1.2
8	**3b**	4.26 ^d^	8.8	32	128	4	4	61 ± 6
9	**3c**	5.96 ^d^	10.0	2000	2000	64	64	37 ± 5
10	Alamethicin [41]	--	--			16	--	62.5 ^f^ [42]

^a^ Average of values calculated using VCCLab and Molinspiration software. ^b^ Retention time in HPLC C18 reverse phase, CH_3_CN/H_2_O 70/30 (0.1% HCO_2_H). ^c^ Value for the protonated forms. ^d^ Calculated for the imidazolium cations. ^e^ MIC/MBC values were obtained from a minimum of three separate experiments. Please refer to Supporting Information for MIC/MBC/IC _50_ values in μM. ^f^ Cytotoxicity against MRC-5 cells.

**Table 2:** Estimated critical aggregation concentration (CAC) values obtained in aqueous and bacterial cell culture medium using fluorescence spectroscopy at 25 °C.

Entry	Amphiphilic Compound	CAC Fluorescence (mM) ^a^		
		W ^b^	CCM:W ^c^	
			CAC1	CAC2
1	**1a**	0.085	0.004	0.045
2	**1b**	0.084	0.006	0.048
3	**1c**	0.010	0.016	0.21
4	**2a**	4.31	2.89	5.4
5	**2b**	4.57	3.46	8.4
6	**2c**	2.34	2.25	3.54
7	**3a**	0.033	0.018	0.325
8	**3b**	0.098	0.063	0.33
9	**3c**	nd ^d^	nd ^d^	

^a^ CAC values from the break point. ^b^ In water. ^c^ In the 1/1 bacterial cell culture medium/water. ^d^ Low solubility.

## Supplementary Materials

The following are available online at https://www.mdpi.com/1424-8247/13/12/482/s1.
