# Supplementary material for: Imidazole and Imidazolium Antibacterial Drugs Derived from Amino Acids
Source: Pharmaceuticals (Basel). 2020 Dec 21;13(12):482. doi: 10.3390/ph13120482 (PMC7767166; doi:10.3390/ph13120482)
Supplement: Supplementary file 1 [file pharmaceuticals-13-00482-s001.pdf]

Imidazole and Imidazolium Bioactive Drugs Derived from Amino Acids

A. Valls,[1] J. Juan Andreu,[1] E. Falomir,[1] S.V. Luis,[1] E. Atrián-Blasco,*[2,3] S. G. Mitchell[2,3]* and B. Altava[1]*

[1] Departamento de Química Inorgánica y Orgánica, Universitat Jaume I, Av. Sos Baynat s/n, 12071, Castellón, Spain.

[2] Instituto de Nanociencia y Materiales de Aragón (INMA), Consejo Superior de Investigaciones Científicas-Universidad de Zaragoza, 50009 Zaragoza, Spain.

[3] CIBER de Bioingeniería, Biomateriales y Nanomedicina, Instituto de Salud Carlos III, 28029 Madrid, Spain.

* Correspondence: altava@uji.es and scott.mitchell@csic.es

Fig. S1-S2 SEM images of bacteria after incubation with **1c** and **3b**

Table S1. MIC, MBC and IC_{50} (μM) values

Fig. S3. Correlation between LogP and the retention time for the different compounds

Fig. S4-S8. HPLC chromatograms for compounds **1a-c**, **2a-c**, **3a-c**

Fig. S9-S14. Fluorescence studies of the aggregation of the compounds in different media

Fig. S15. Optical microscopy images for **1a**, **1b** and **1c** at 25 °C

Fig S16. Optical microscopy images for **2a**, **2b** and **2c** at 25 °C

Fig S17. Optical microscopy images for a **3a** and **3b** at 25 °C.

Fig S18. SEM images for **1c** in water**,** in 1/1 water/ bacterial culture and in bacterial culture media.

Fig. S19. Absorbance variation at 600 nm against time for **1a** in water, 1/1 bacterial cell culture/water and bacterial cell culture media.

Fig. S20. Absorbance variation at 600 nm against time for **1c** in water, 1/1 bacterial cell culture/water and bacterial cell culture media.

Fig. S21. ^1H NMR (400 MHz) of **3a** in $CDCl_3$ and CD_3OD.

Fig. S22. ^{13}C NMR (101 MHz) of **3a** in $CDCl_3$.

Fig. S23. MS (ESI) of **3a**.

Fig. S24. ^1H NMR spectrum of **3b** in $CDCl_3$ (300 MHz).

Fig. S25. ^{13}C NMR spectrum of **3b** in $CDCl_3$ (101 MHz)

Fig. S26. MS (ESI) of **3b**.

Fig. S27. ^1H NMR spectrum of **3c** in $CDCl_3$ (300 MHz).

Fig. S28. ^{13}C NMR spectrum of **3c** in $CDCl_3$ (101 MHz).

Fig. S29. MS (ESI) of **3c**.

Fig. S30. ^{1}H NMR of **2b** in $CDCl_3$. (400 MHz).

Fig. S31. ^{13}C NMR of **2b** in $CDCl_3$. (101 MHz).

Fig. S32. MS (ESI) of **2b**.

Fig. S33. ^{1}H NMR spectrum of **2c** in $CDCl_3$ (500 MHz).

Fig. S34. ^{13}C NMR spectrum of **2c** in $CDCl_3$ (126 MHz).

Fig. S35. MS (ESI) of **2c**.

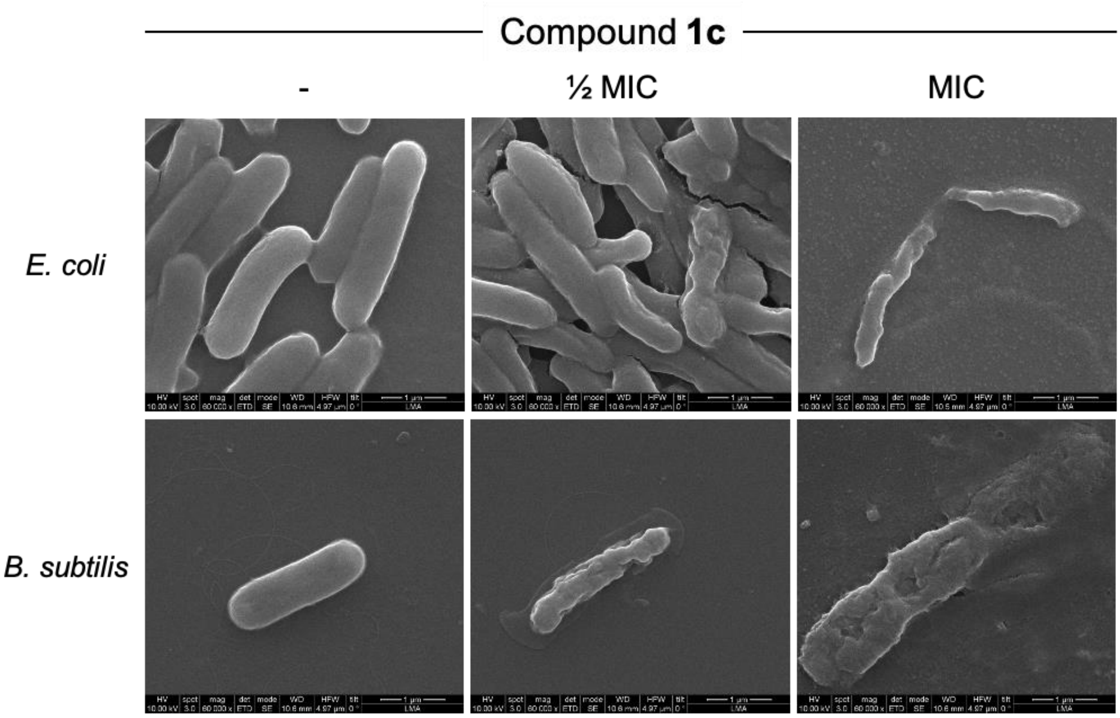

Fig. S1. Scanning Electron Microscopy (SEM) images of *E. coli* and *B. subtilis* bacteria without treatment (-) and after incubation with compound **1c** at its ½ MIC and MIC (60000 x).

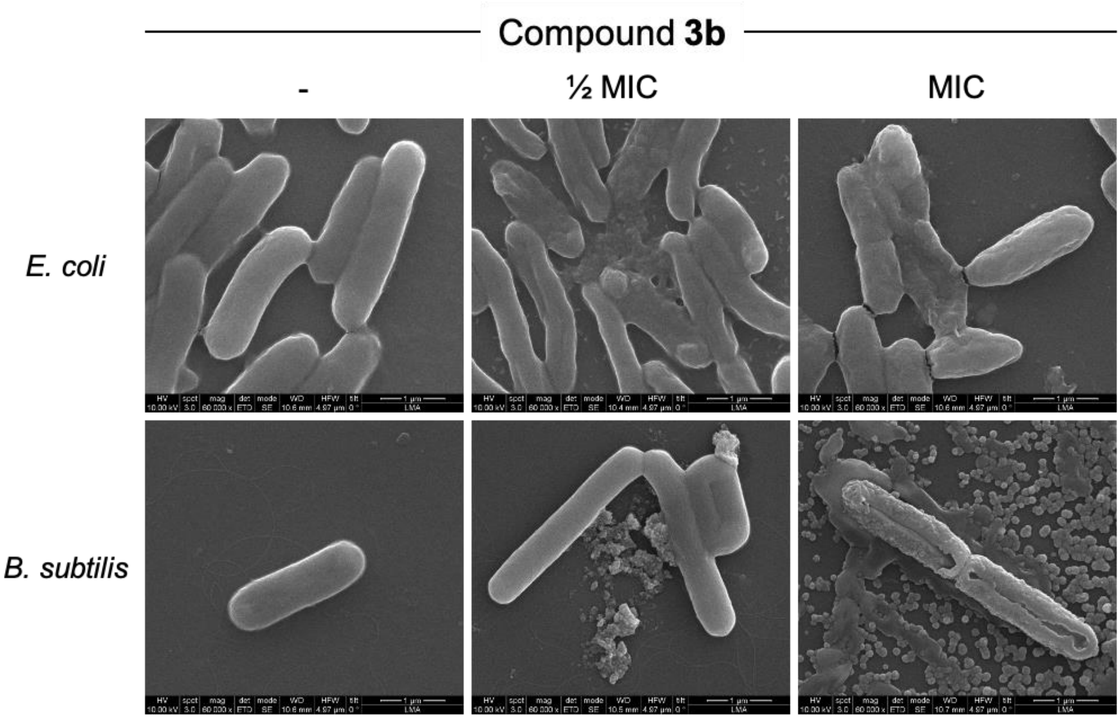

Fig S2. Scanning Electron Microscopy (SEM) images of *E. coli* and *B. subtilis* bacteria without treatment (-) and after incubation with compound **3b** at its ½ MIC and MIC (60000 x).

Table S1. Minimum Inhibitory Concentration (MIC, μM), Minimal Bactericidal Concentration (MBC, μM), the half maximal inhibitory concentration IC_{50} (μM) values

Entry	Compound	*E. coli*		*B. subtilis*		HEK-293
		MIC[a] μM	MBC[a] μM	MIC[a] μM	MBC[a] μM	IC_{50} μM
**1**	**1a**	>6000	>6000	48	48	9.6 ± 1.6
**2**	**1b**	288	581	9	18	1.8 ± 0.4
**3**	**1c**	269	269	19	34	19 ± 4
**4**	**2a**	>9000	>9000	>9000	>9000	>200
**5**	**2b**	>5000	>5000	2591	2591	>200
**6**	**2c**	1444	>2900	180	361	>200
**7**	**3a**	2662	>5000	42	42	19 ± 3
**8**	**3b**	57	229	7	7	109 ± 10
**9**	**3c**	1986	1986	62	62	36 ± 5

[a] Mode – most frequent value - of MIC and MBC values from at least three experiments (when obtaining the same values) or at least five experiments (when the values obtained are different).

a)

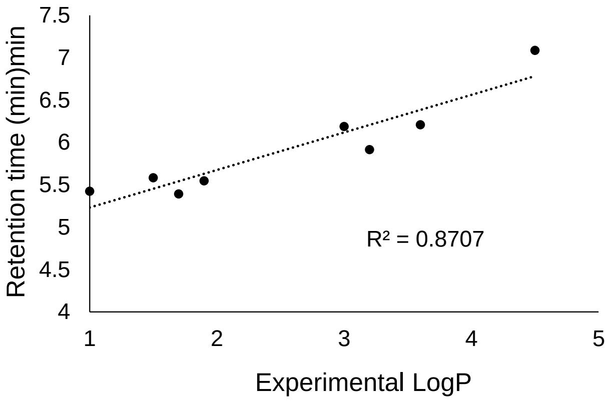

Retention time	log P	Compound
5.392	1.7	Acetophenone
6.188	3	Bromobenzene
5.916	3.2	Benzophenone
6.208	3.6	Naphtalene
7.088	4.5	Phenanthrene
5.424	1	Acetanilide
5.583	1.5	Phenol
5.546	1.9	Benzoic acid

b)

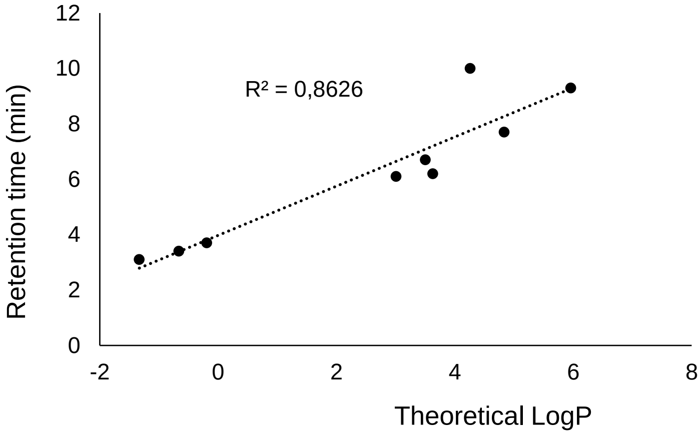

Fig. S3. a) Correlation between experimental LogP values and experimental retention time for different commercial compounds **b)** Correlation between LogP and the retention time for the different compounds **1a-c**, **2a-c**, **3a-c.**

CH_3OH

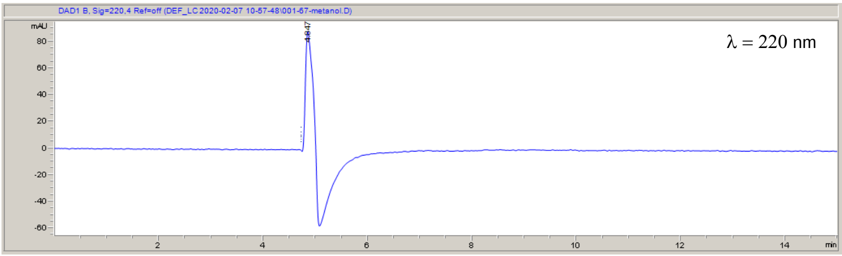

Acetanilide

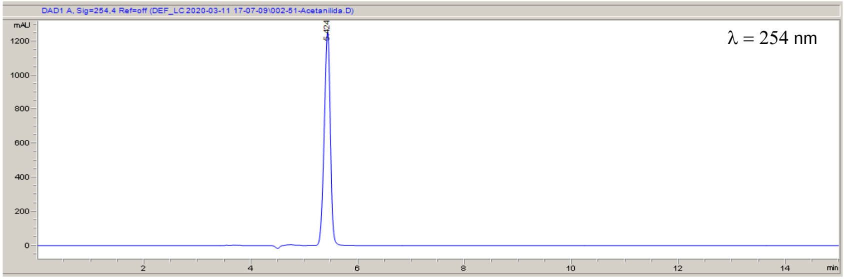

Naphtalene

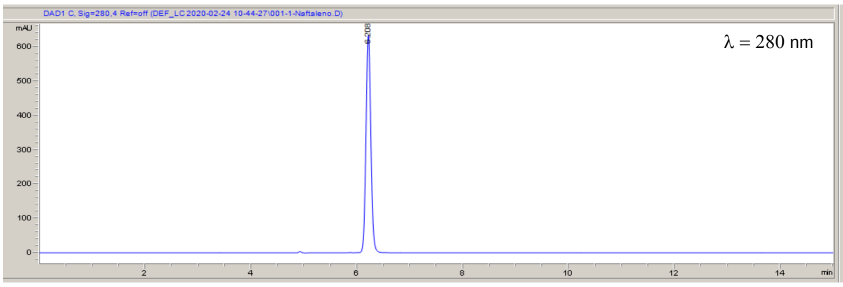

Phenol

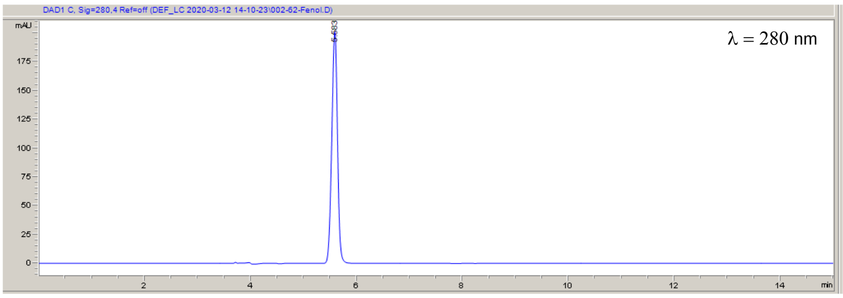

Benzoic acid

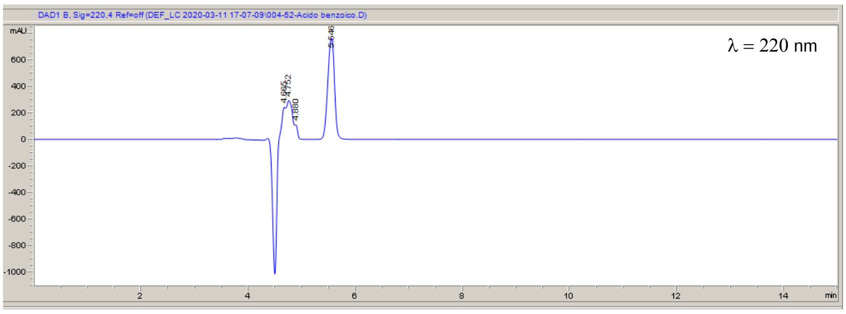

Fig. S4. HPLC chromatograms for methanol, acetanilide, naphthalene, phenol and benzoic acid. Mobile phase water/CH_3CN 30/70 (0.1% HCO_2H), 25°C and flow 0.2 mL/min.

Benzophenone

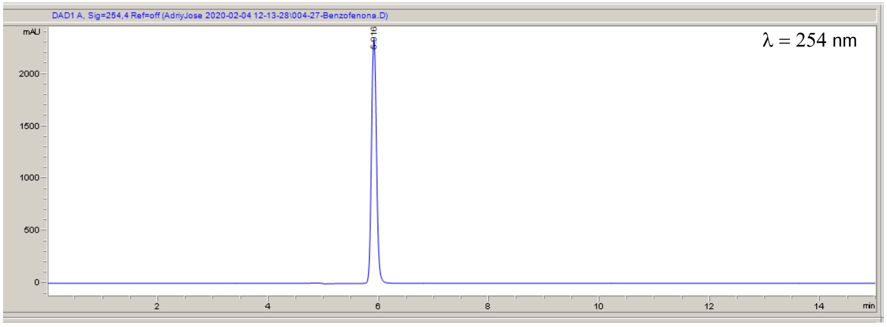

Phenantrene

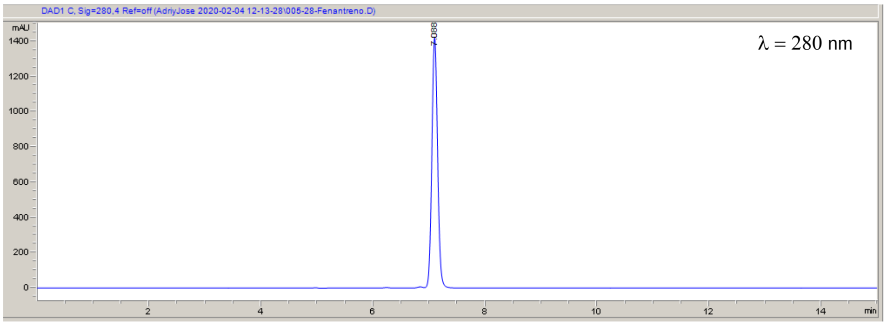

Acetophenone

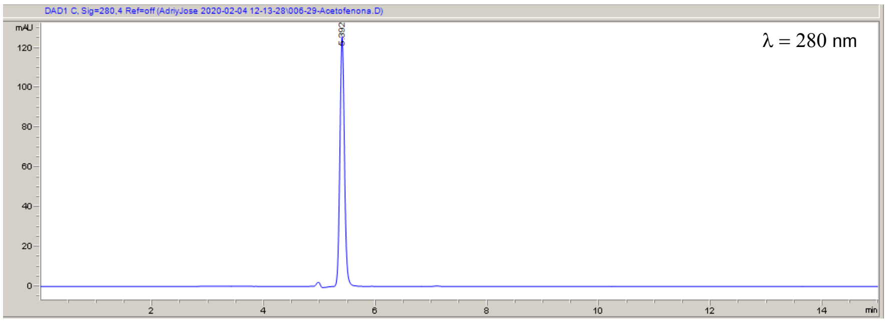

Bromobenzene

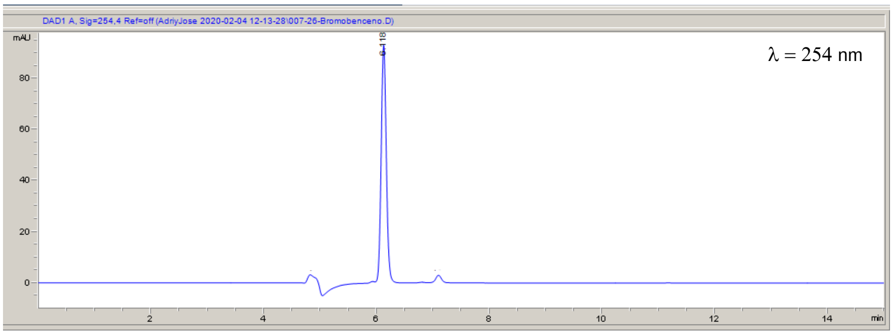

Fig. S5. HPLC chromatograms for benzophenone, phenantrene, acetophenone and bromobenzene. Mobile phase water/CH_3CN 30/70 (0.1% HCO_2H), 25 °C and flow 0.2 mL/min.

1a

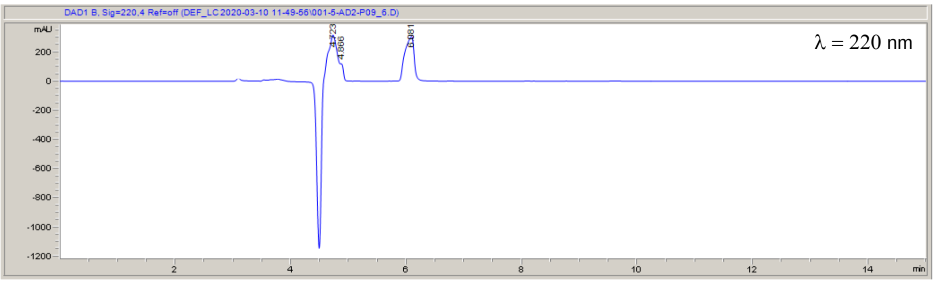

1b

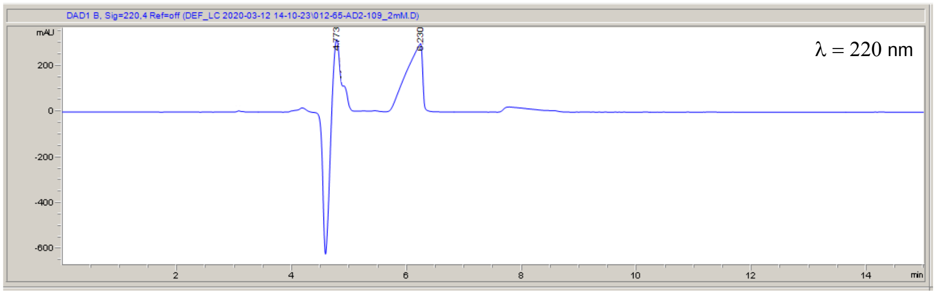

1c

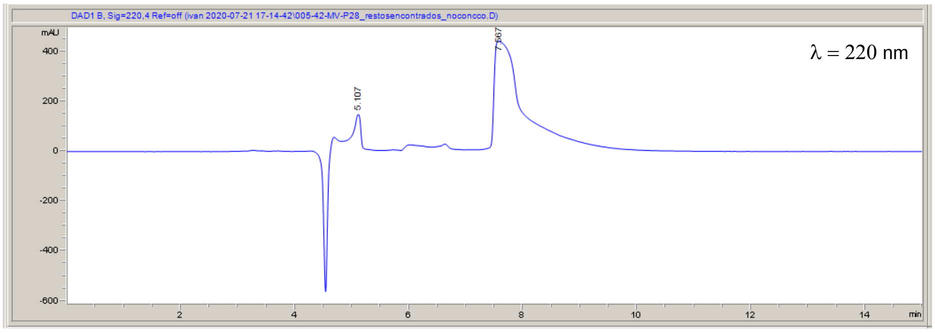

Fig. S6. HPLC chromatograms for compounds **1a-c**. Mobile phase water/CH_3CN 30/70 (0.1% HCO_2H), 25 °C and flow 0.2 mL/min.

2a

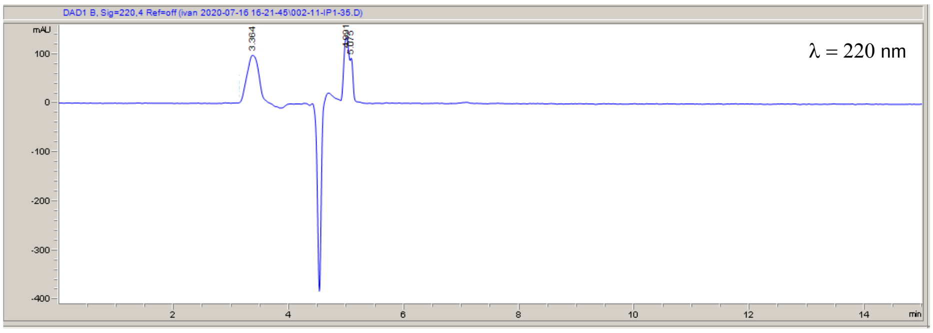

2b

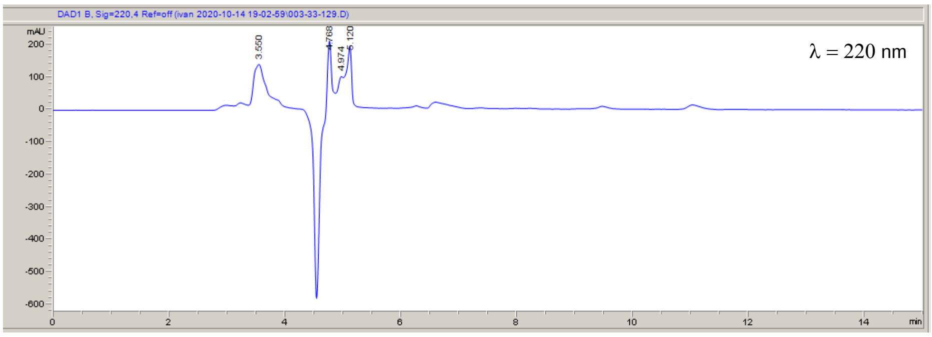

2c

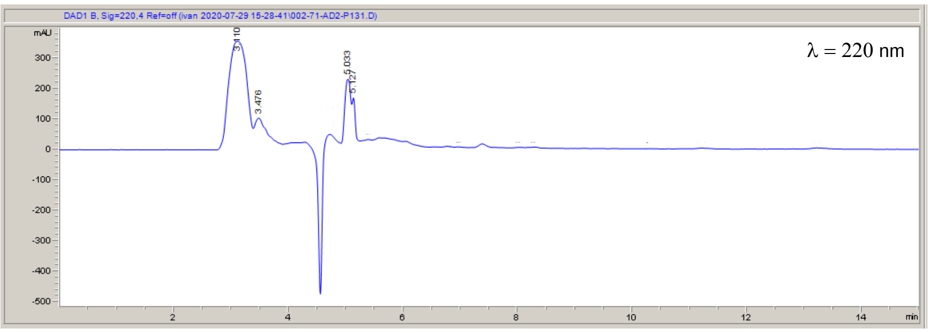

Fig. S7. HPLC chromatograms for compounds **2a-c**. Mobile phase water/CH_3CN 30/70 (0.1% HCO_2H), 25 °C and flow 0.2 mL/min.

3a

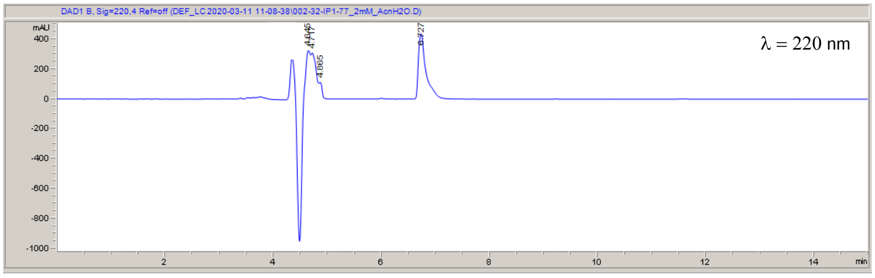

3b

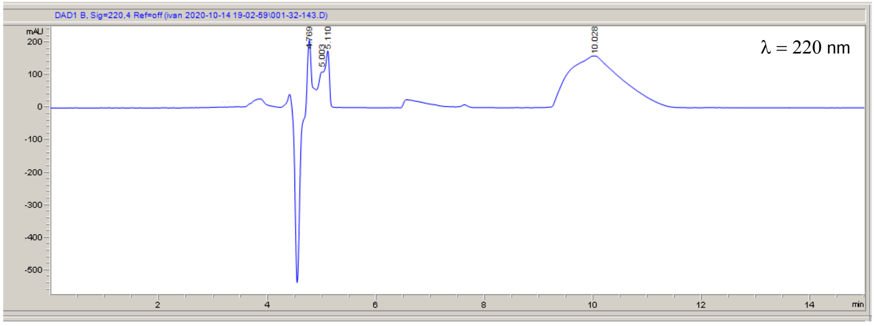

3c

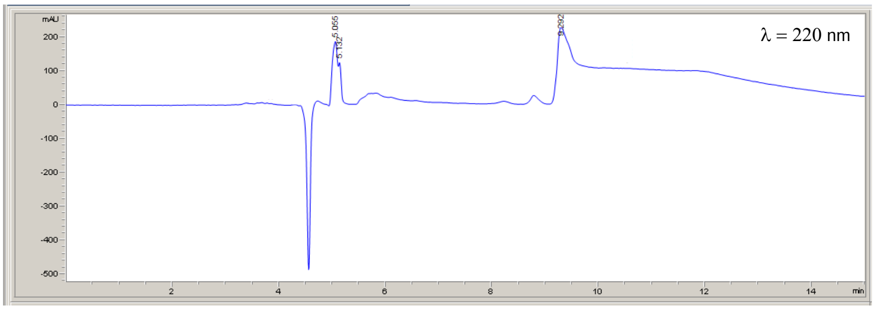

Fig. S8. HPLC chromatograms for compounds **3a-c**. Mobile phase water/CH_3CN 30/70 (0.1% HCO_2H), 25 °C and flow 0.2 mL/min.

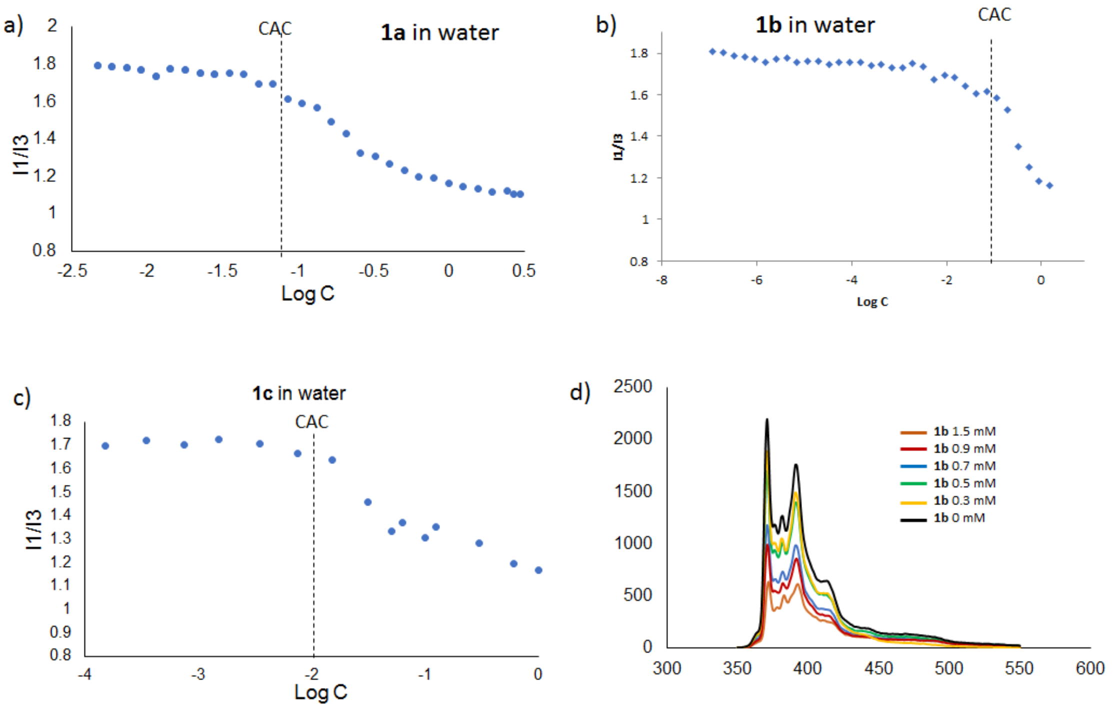

Fig. S9. a) - c) Plots of the I_1/I_3 ratios *v* log C for **1a-1c** in H_2O at 25 °C. d) Emission spectra of pyrene in H_2O at 25 °C in the presence of different amounts of **1b**.

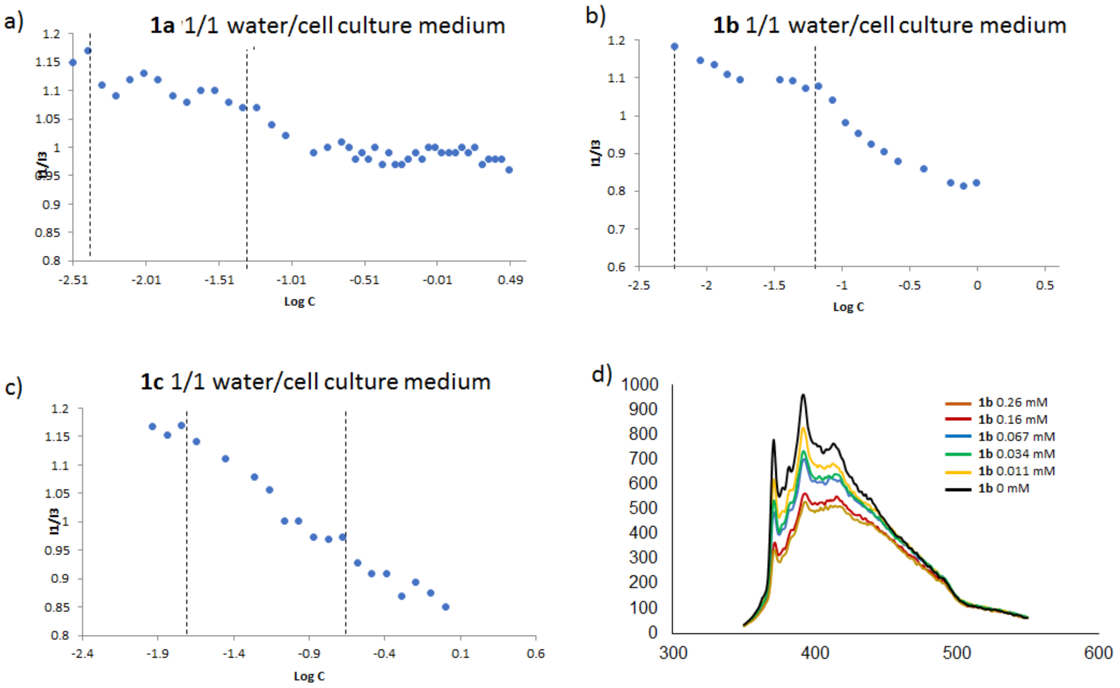

Fig. S10. a) - c) Plots of the I_1/I_3 ratios *v* log C for **1a-1c** in 1/1 water/bacterial cell culture medium at 25 °C. d) Emission spectra of pyrene in 1/1 water/bacterial cell culture medium at 25 °C in the presence of different amounts of **1b**.

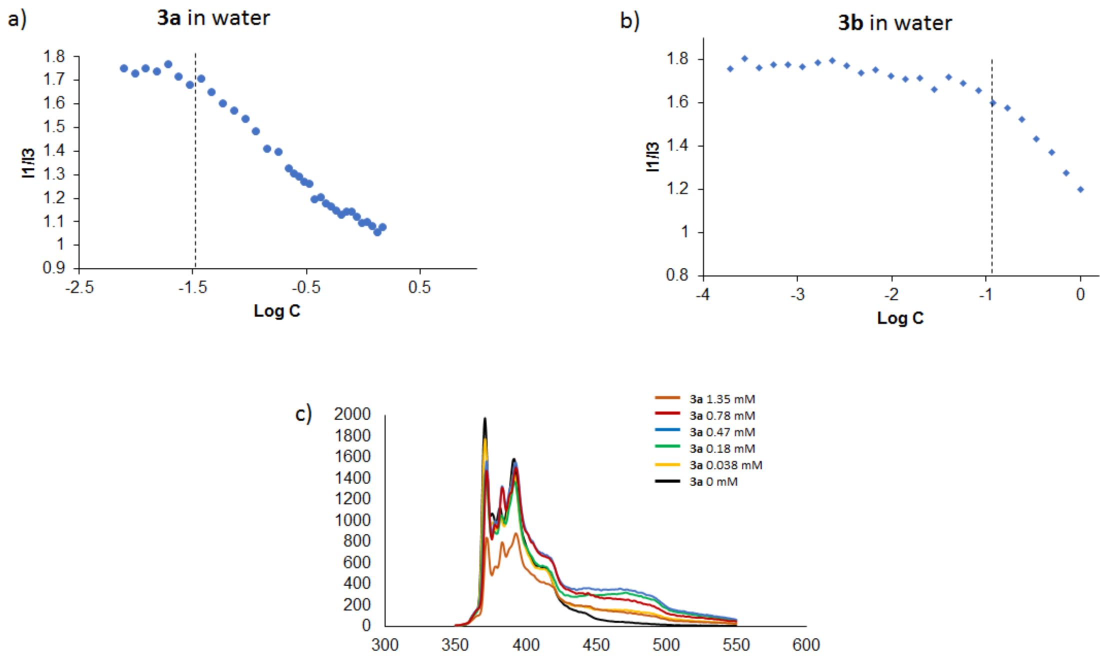

Fig. S11. a)-b) Plots of the I_1/I_3 ratios *v* log C for **3a-3b** in H_2O at 25 °C. d) Emission spectra of pyrene in H_2O at 25 °C in the presence of different amounts of **3a**.

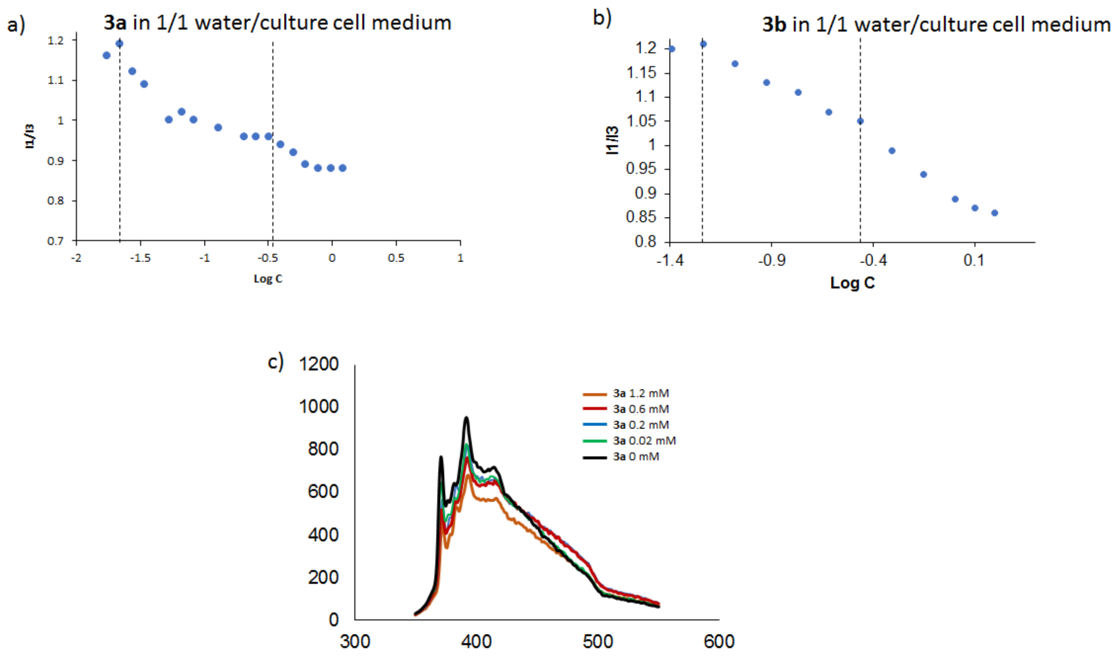

Fig. S12. a)-b) Plots of the I_1/I_3 ratios *v* log C for **3a-3b** in 1/1 water/bacterial cell culture medium at 25 °C. d) Emission spectra of pyrene in 1/1 water/bacterial cell culture medium at 25 °C in the presence of different amounts of **3a**.

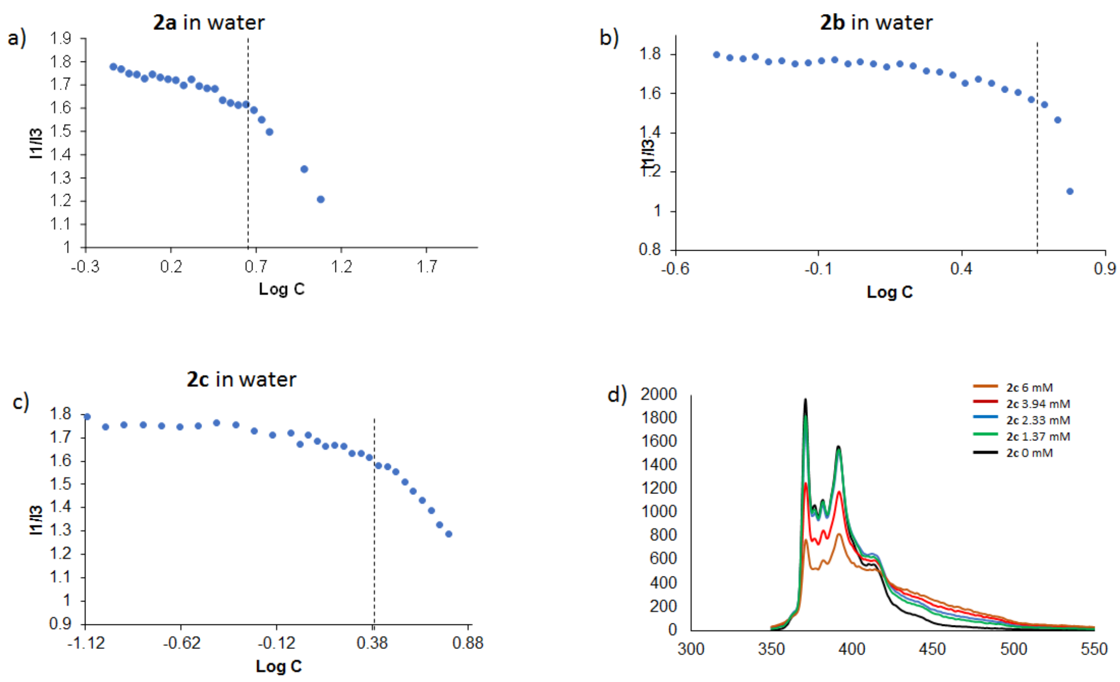

Fig. S13. a) - c) Plots of the I_1/I_3 ratios *v* log C for **2a-2c** in H_2O at 25 °C. d) Emission spectra of pyrene in H_2O at 25 °C in the presence of different amounts of **2c**.

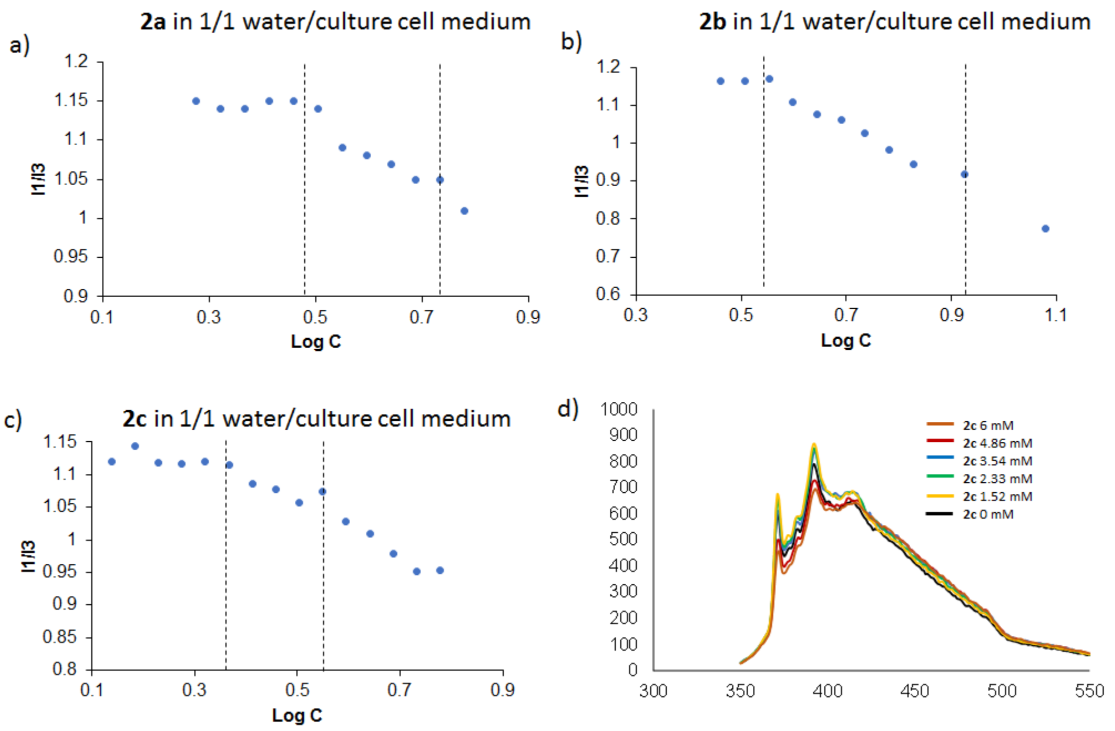

Fig. S14. a) - c) Plots of the I_1/I_3 ratios *v* log C for **2a-2c** in 1/1 water/ bacterial cell culture medium at 25 °C. d) Emission spectra of pyrene in 1/1 water/ bacterial cell culture medium at 25 °C in the presence of different amounts of **2c**.

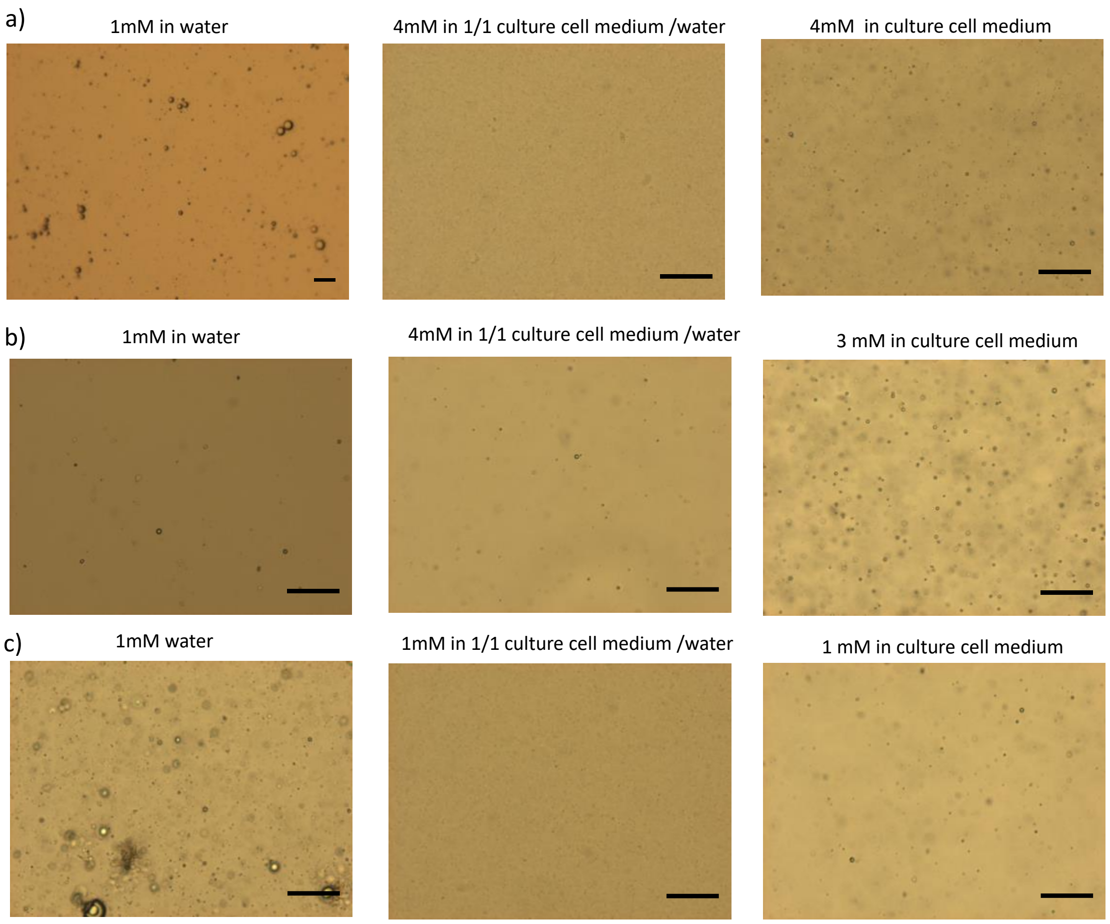

Fig. S15. Optical microscopy images for a) **1a** b) **1b** and c) **1c** at 25 °C in water, 1/1 water/bacterial cell culture medium and bacterial culture medium. Scale bar 50 µm.

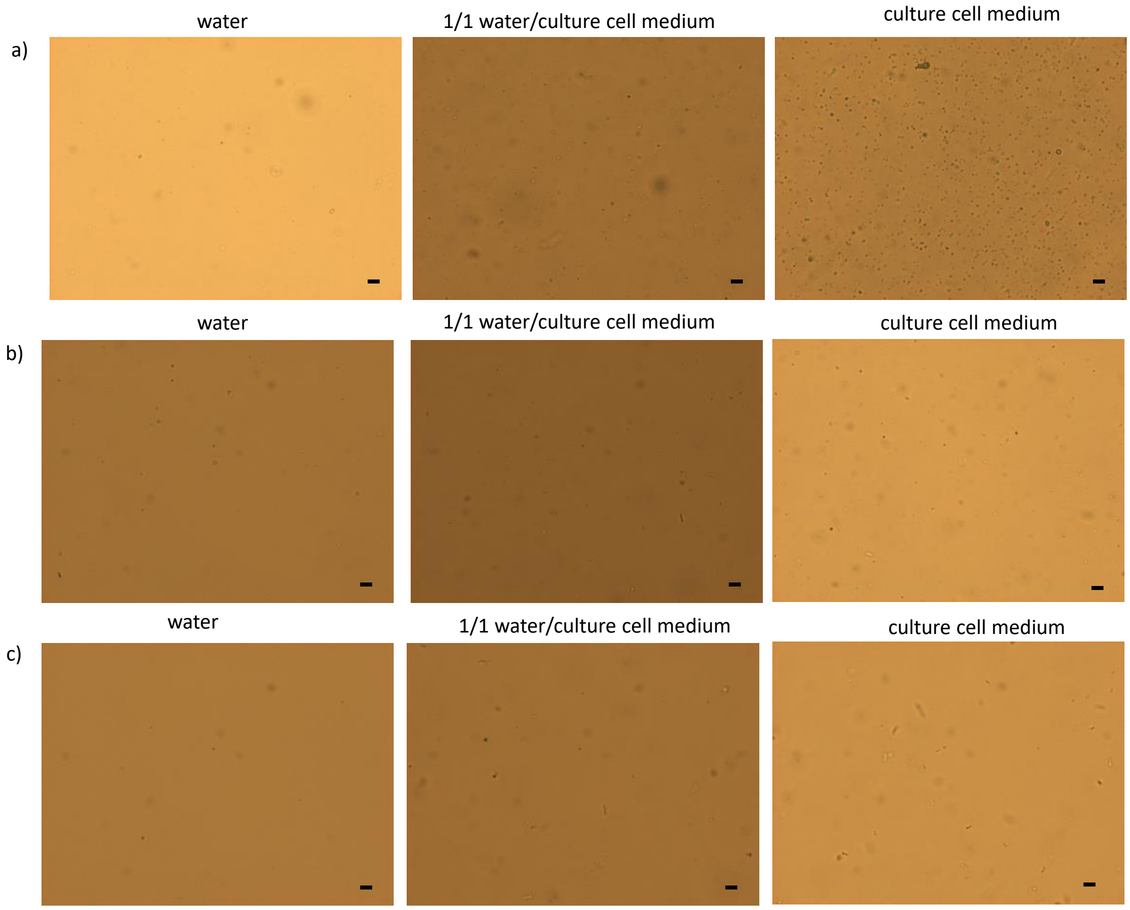

Fig S16. Optical microscopy images for a) **2a** b) **2b** and c) **2c** 4 mM at 25 ºC, in water, 1/1 water/bacterial cell culture medium and bacterial cell culture medium. Scale bar 10 μM.

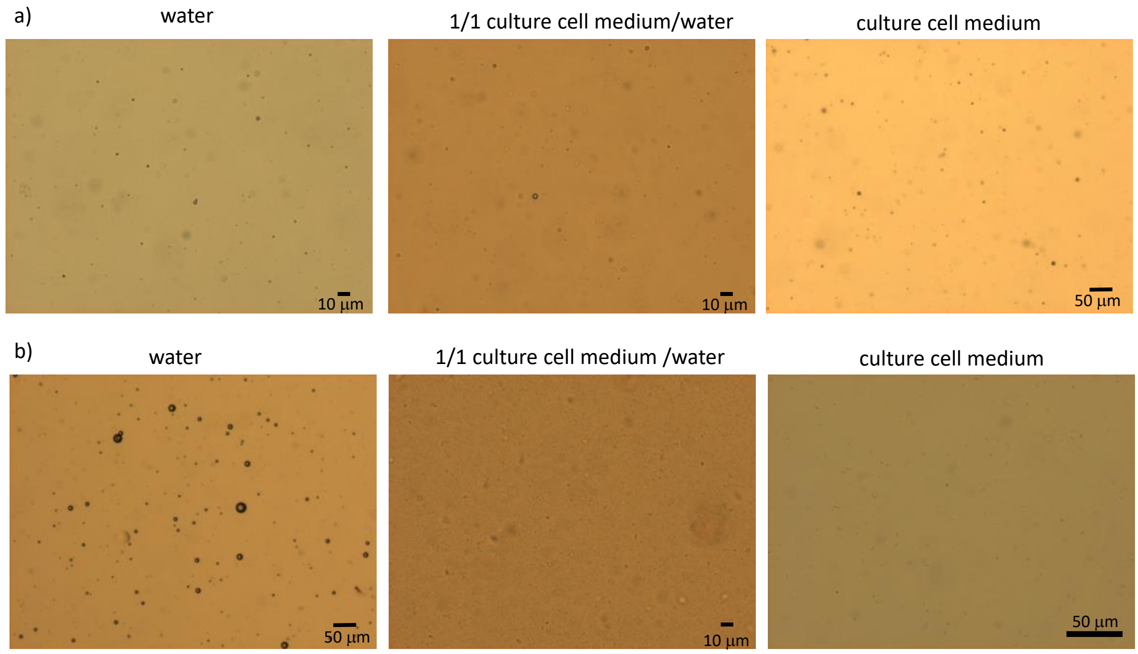

Fig S17. Optical microscopy images for a) **3a** 0.5 mM and b) **3b** 0.5 mM at 25 ºC, in water, 1/1 water/bacterial cell culture medium and bacterial cell culture medium.

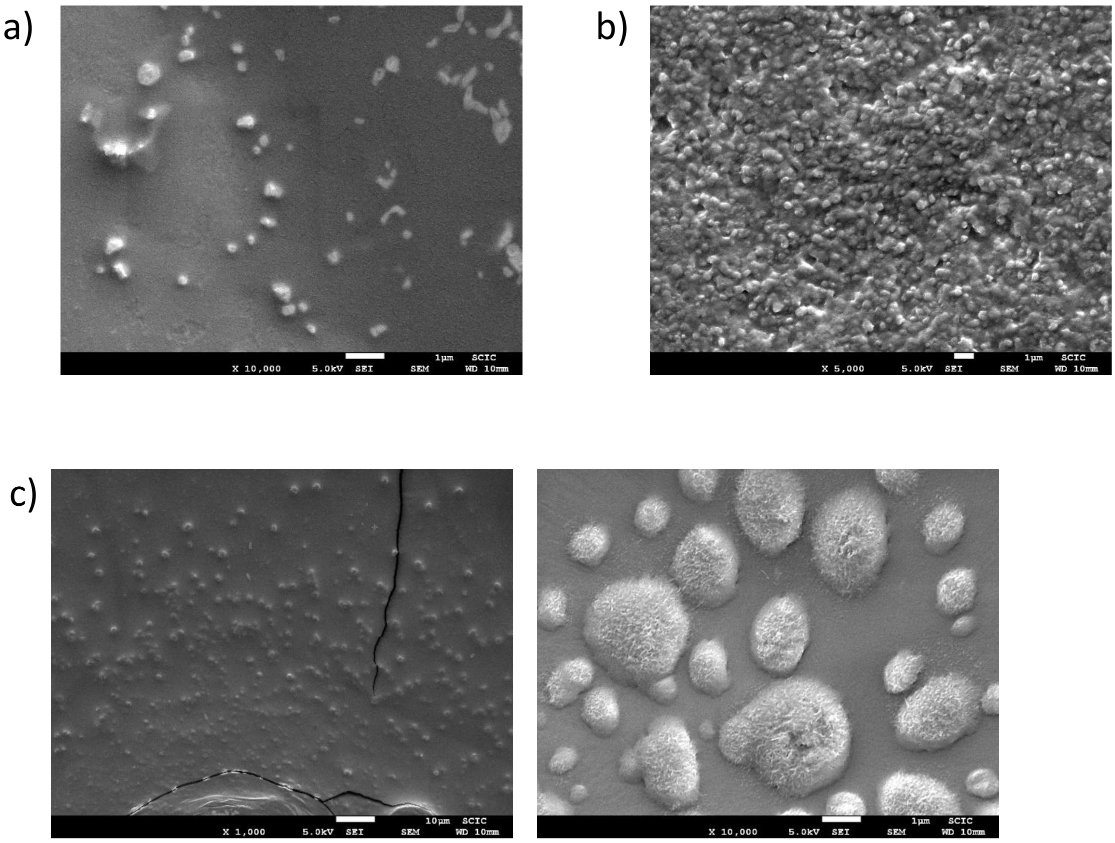

Fig S18. SEM images for **1c** a) 0.5 mM in water**;** b) 1 mM in 1/1 water/bacterial cell culture medium and c) 1 mM in bacterial cell culture medium.

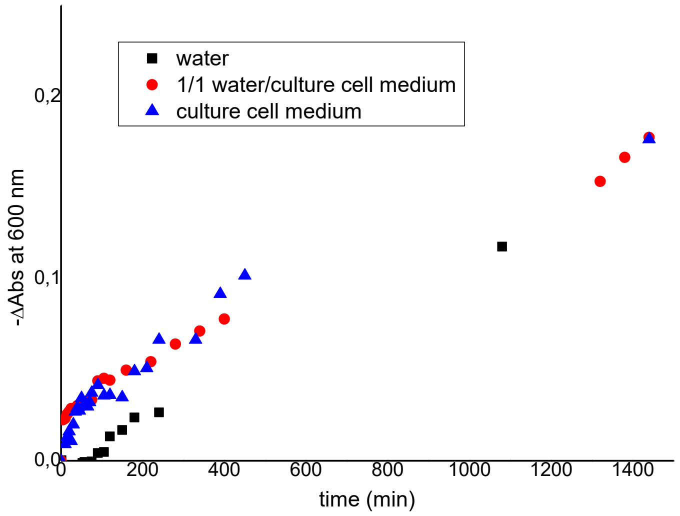

Fig. S19. Absorbance variation at 600 nm against time for **1a** (1 mM) in water, 1/1 water/bacterial cell culture medium, and bacterial cell culture medium.

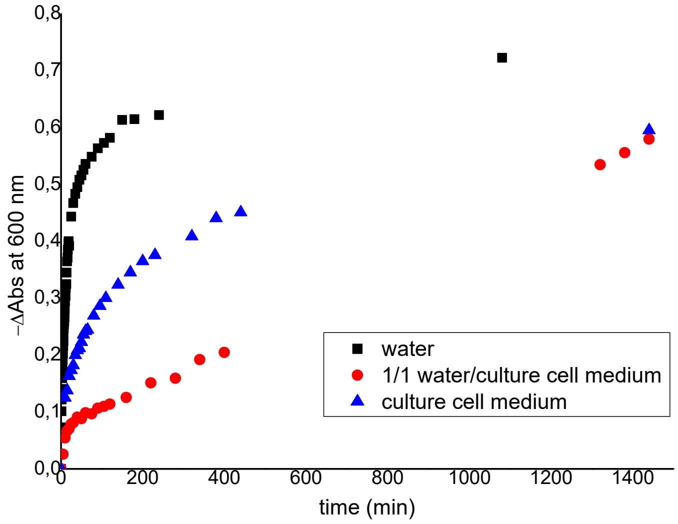

Fig. S20. Absorbance variation at 600 nm against time for **1c** (1mM) in water, 1/1 water/bacterial cell culture medium and bacterial cell culture medium.

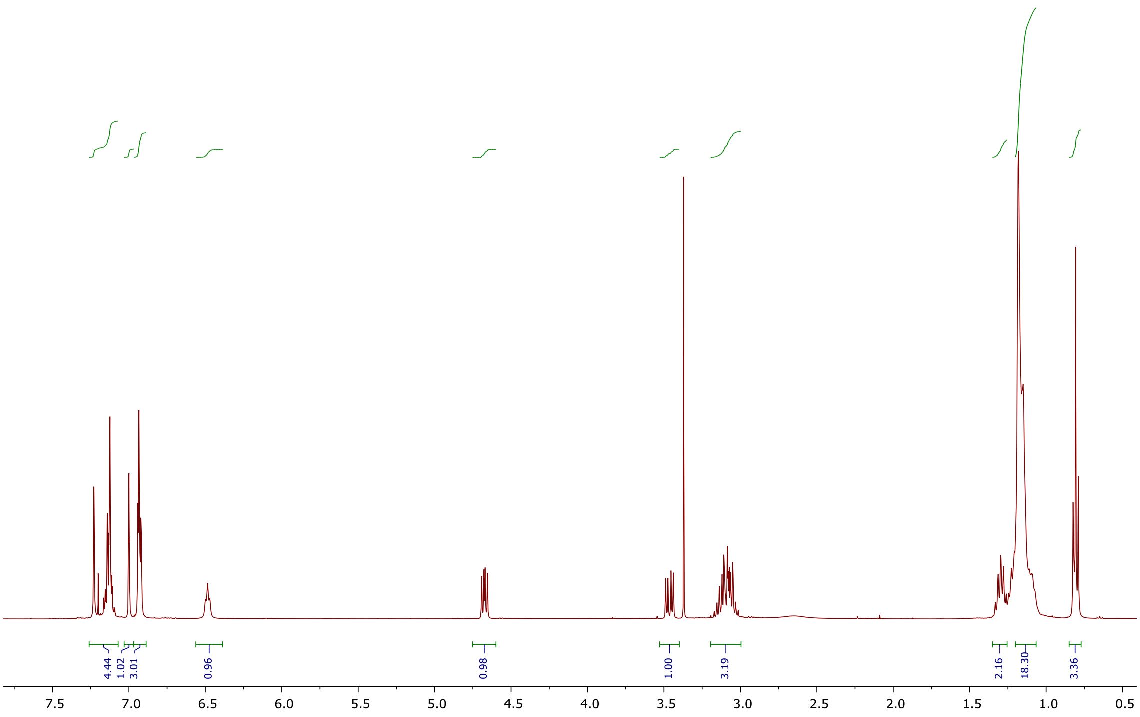

Fig. S21. ^{1}H NMR (400 MHz) of **3a** in $CDCl_3$ and CD_3OD.

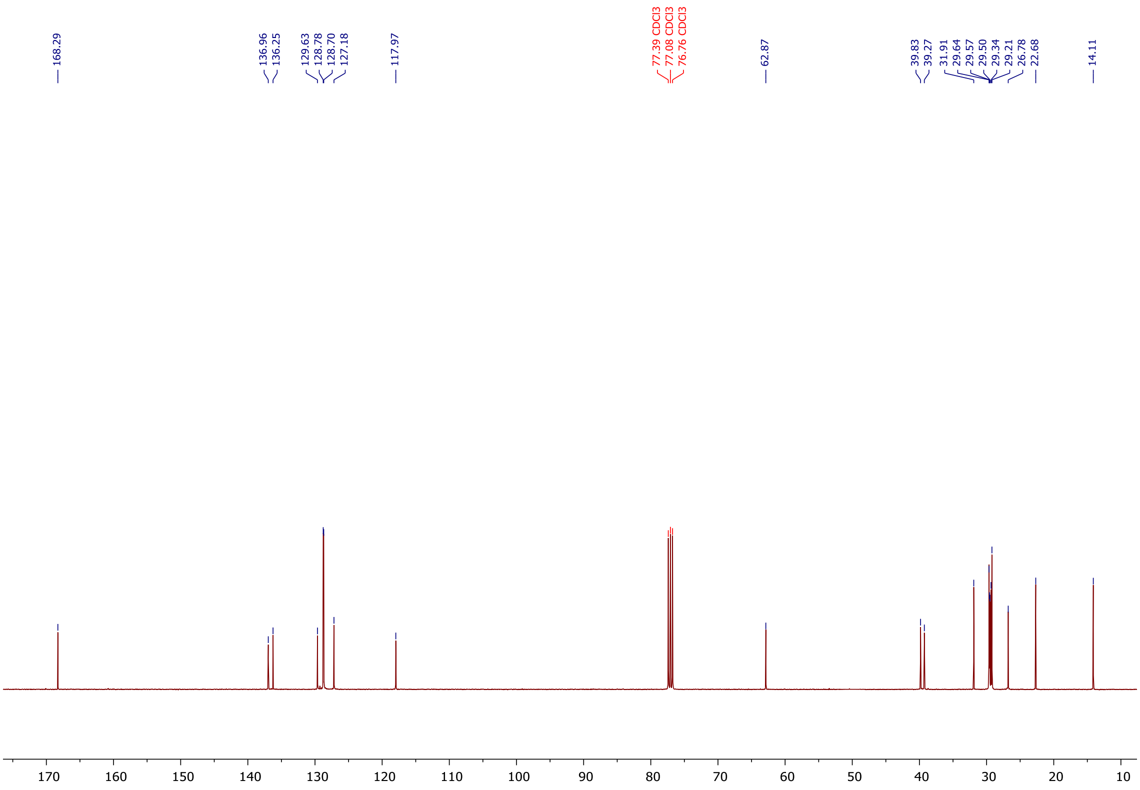

Fig. S22. ^{13}C NMR (101 MHz) of **3a** in $CDCl_3$.

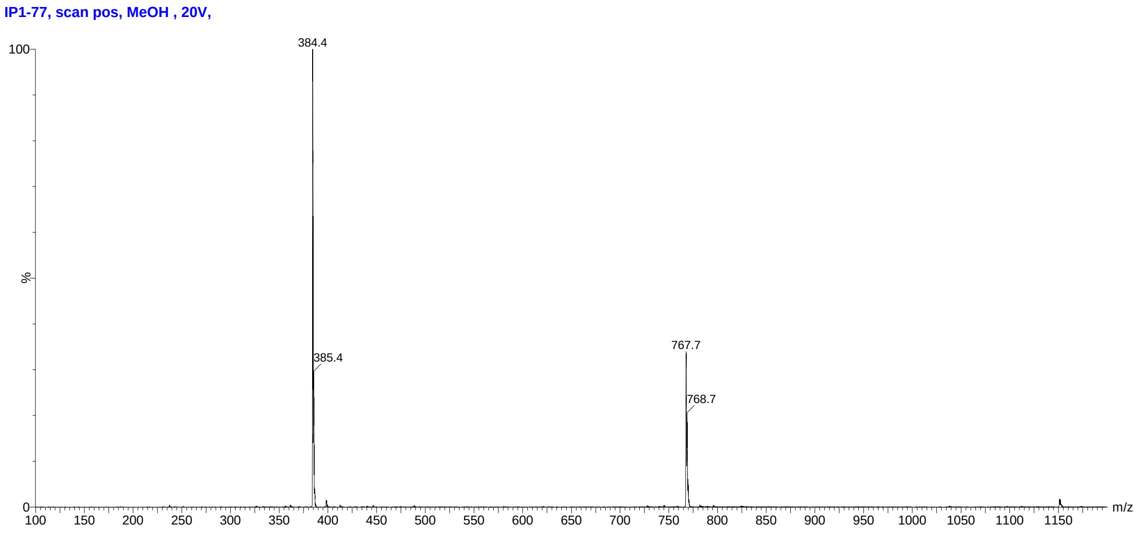

Fig. S23. MS (ESI) of **3a**.

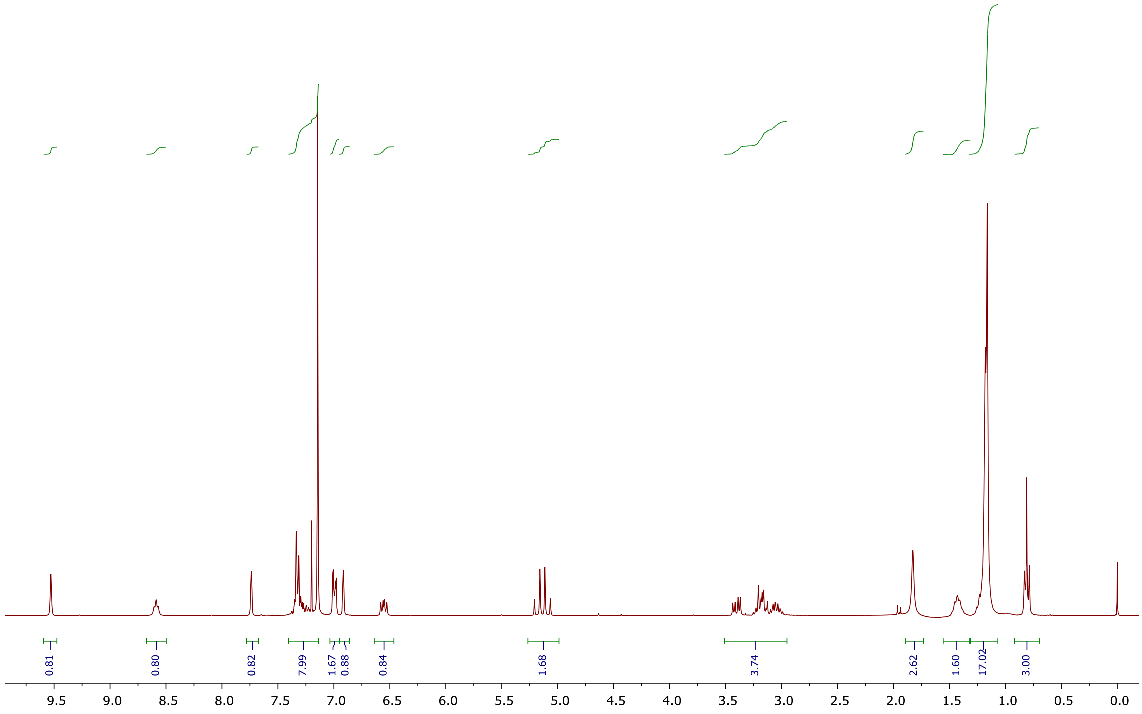

Fig. S24. ^{1}H NMR (300 MHz) of **3b** in $CDCl_3$.

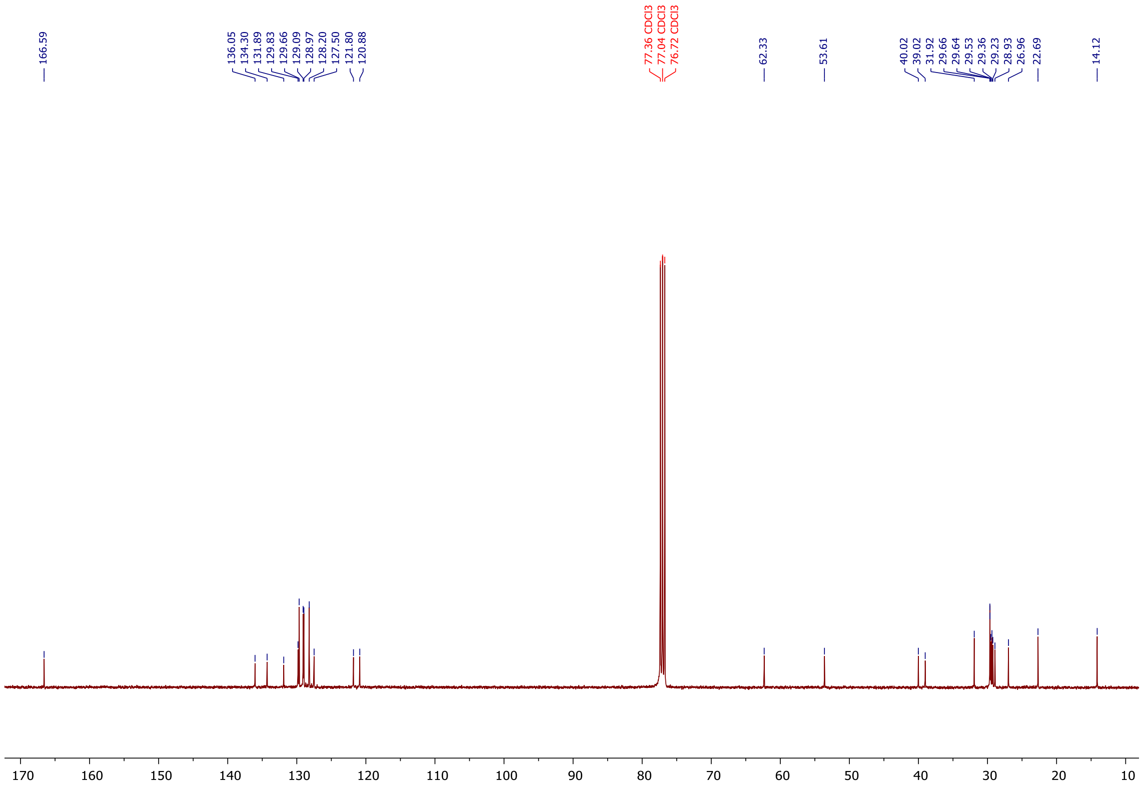

Fig. S25. ^{13}C NMR (101 MHz) of **3b** in $CDCl_3$.

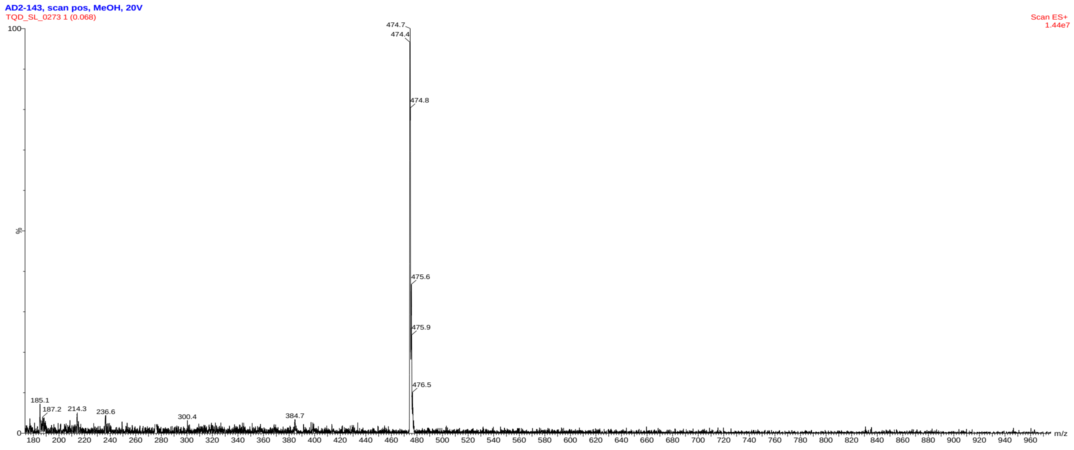

Fig. S26. MS (ESI) of **3b**.

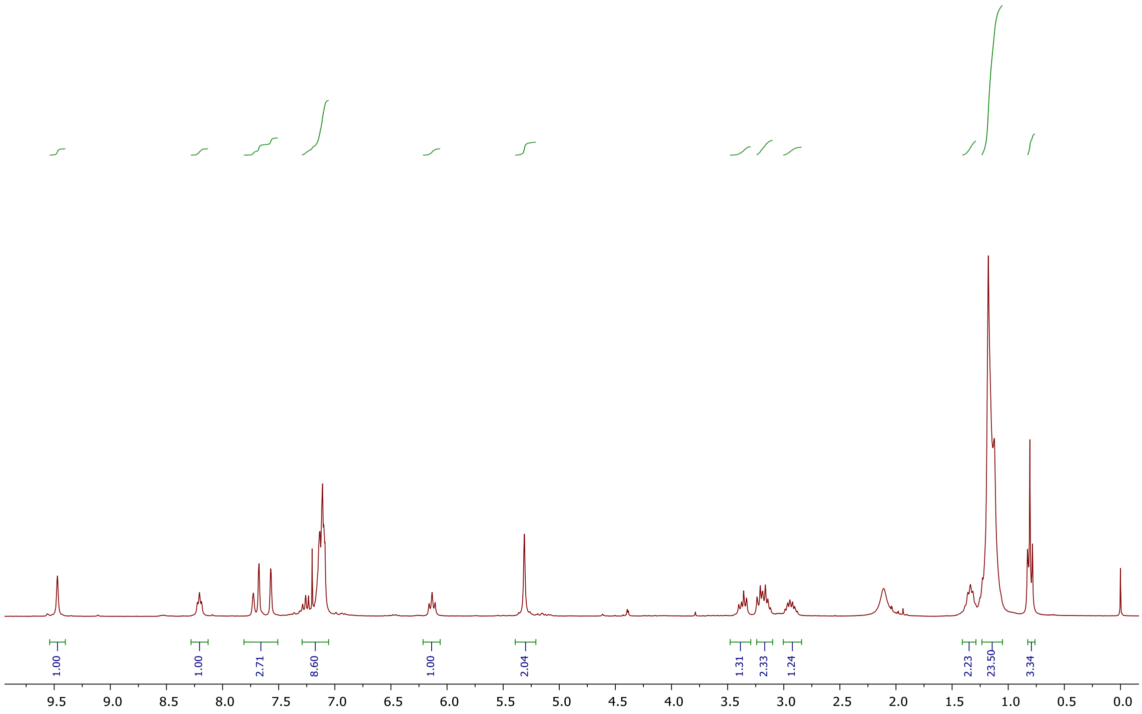

Fig. S27. ^{1}H NMR (300 MHz) of **3c** in $CDCl_3$.

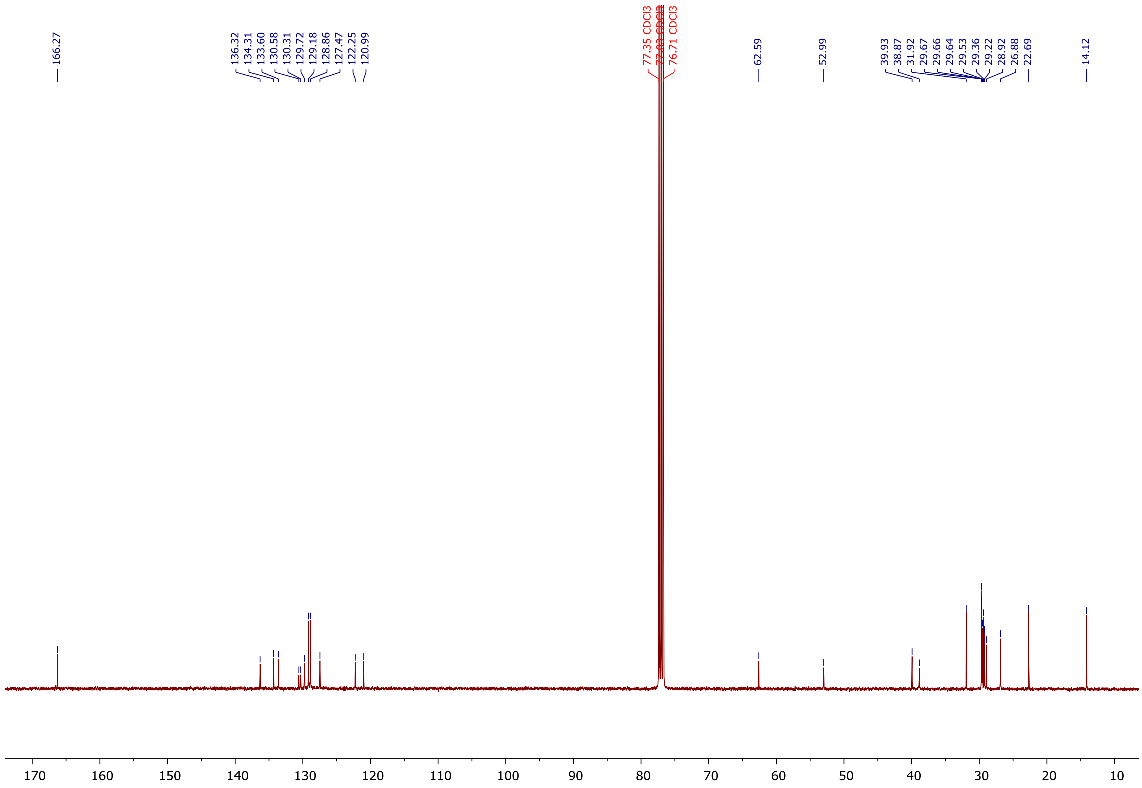

Fig. S28. ^{13}C NMR (101 MHz) of **3c** in $CDCl_3$.

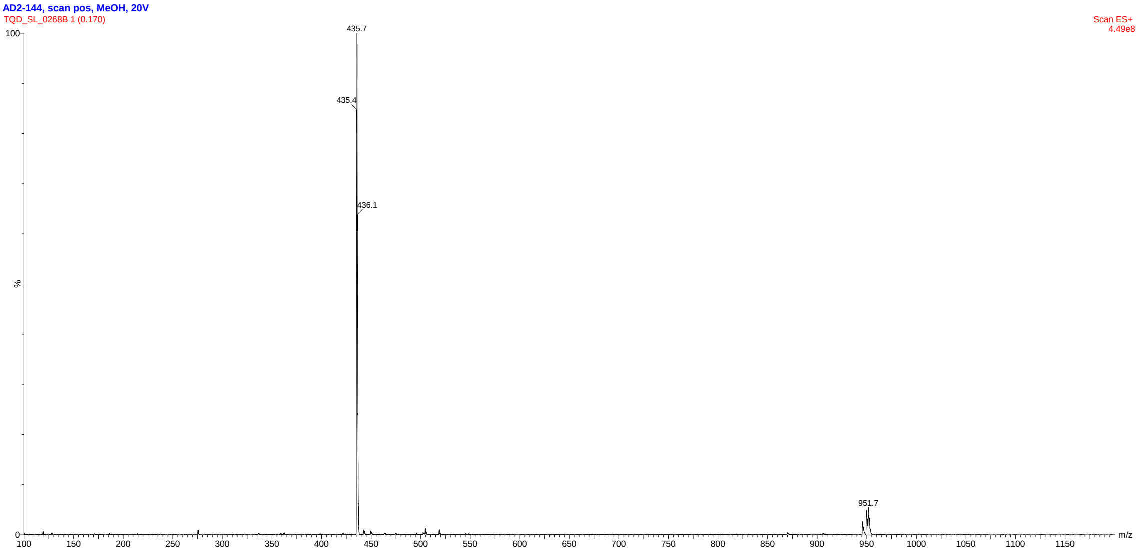

Fig. S29. MS (ESI) of **3c**.

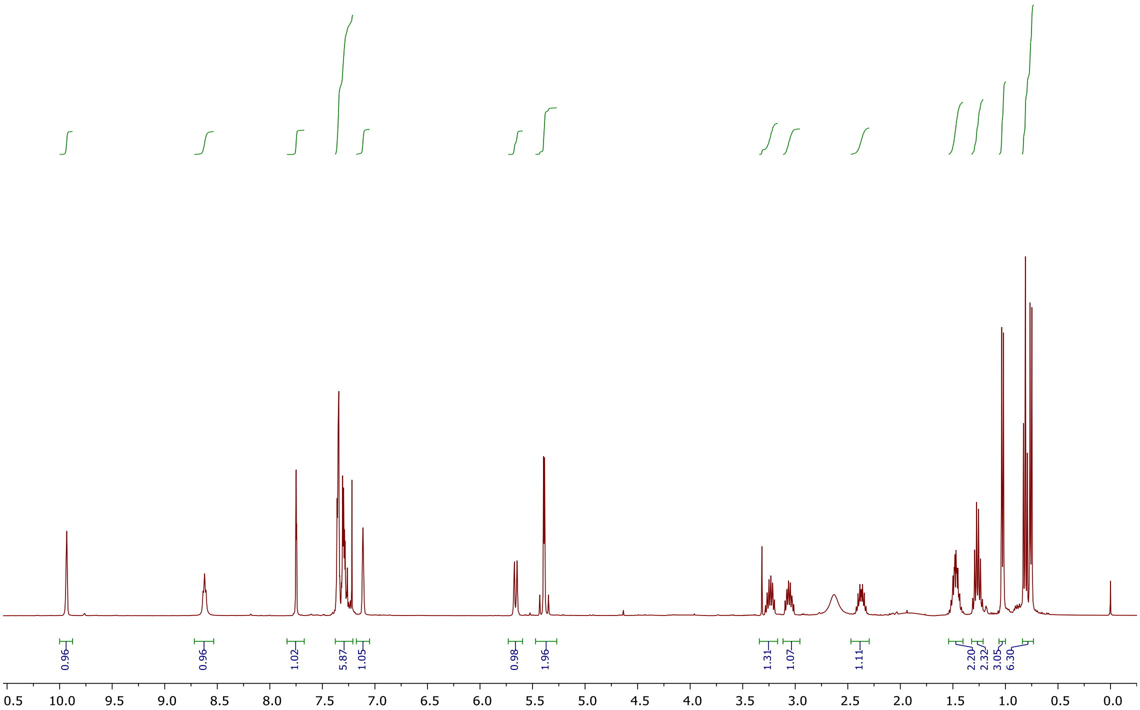

Fig. S30. ^1H NMR (400 MHz) of **2b** in $CDCl_3$.

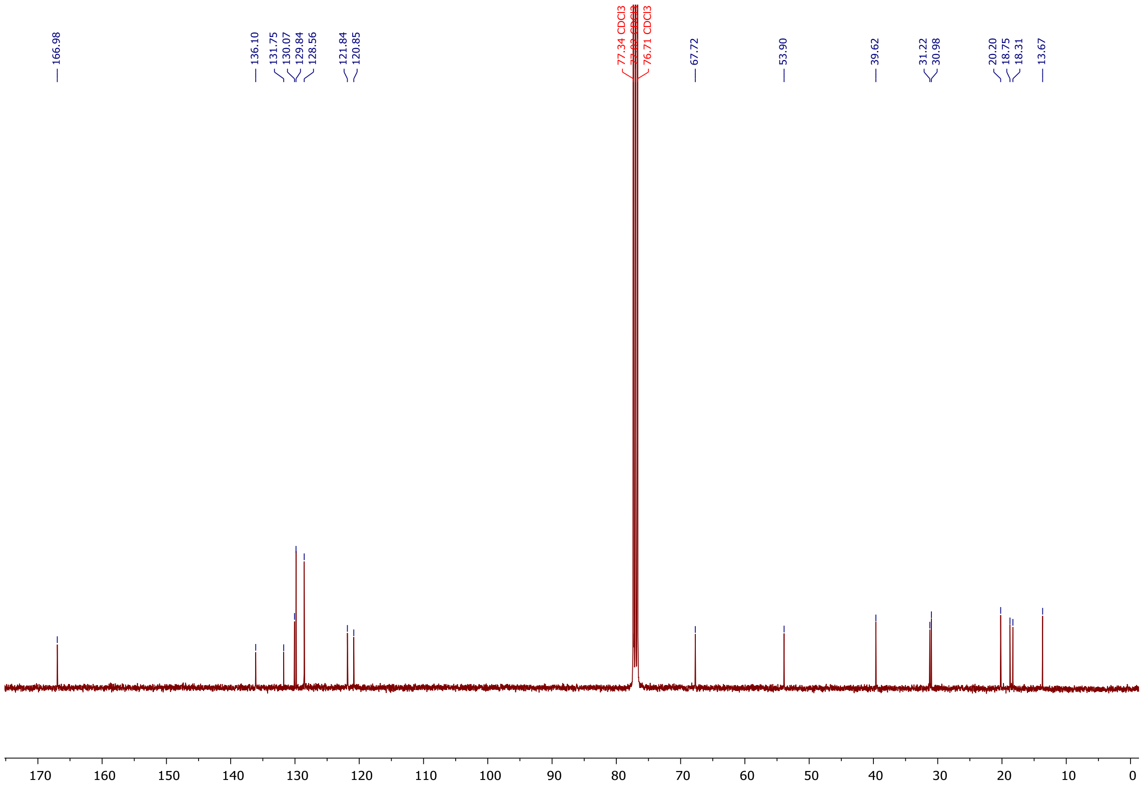

Fig. S31. ^{13}C NMR (101 MHz) of **2b** in $CDCl_3$.

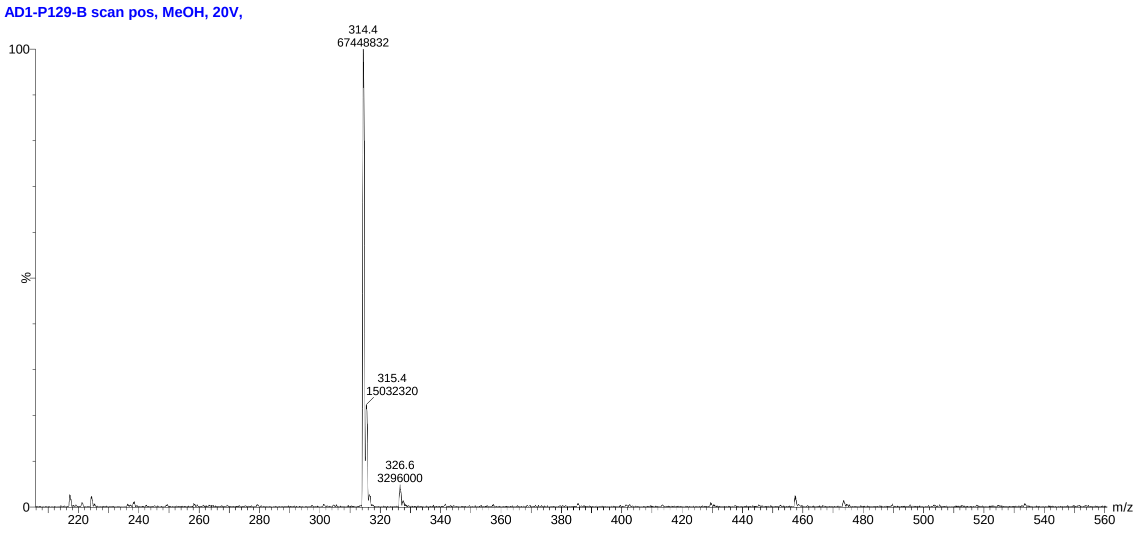

Fig. S32. MS (ESI) of **2b**.

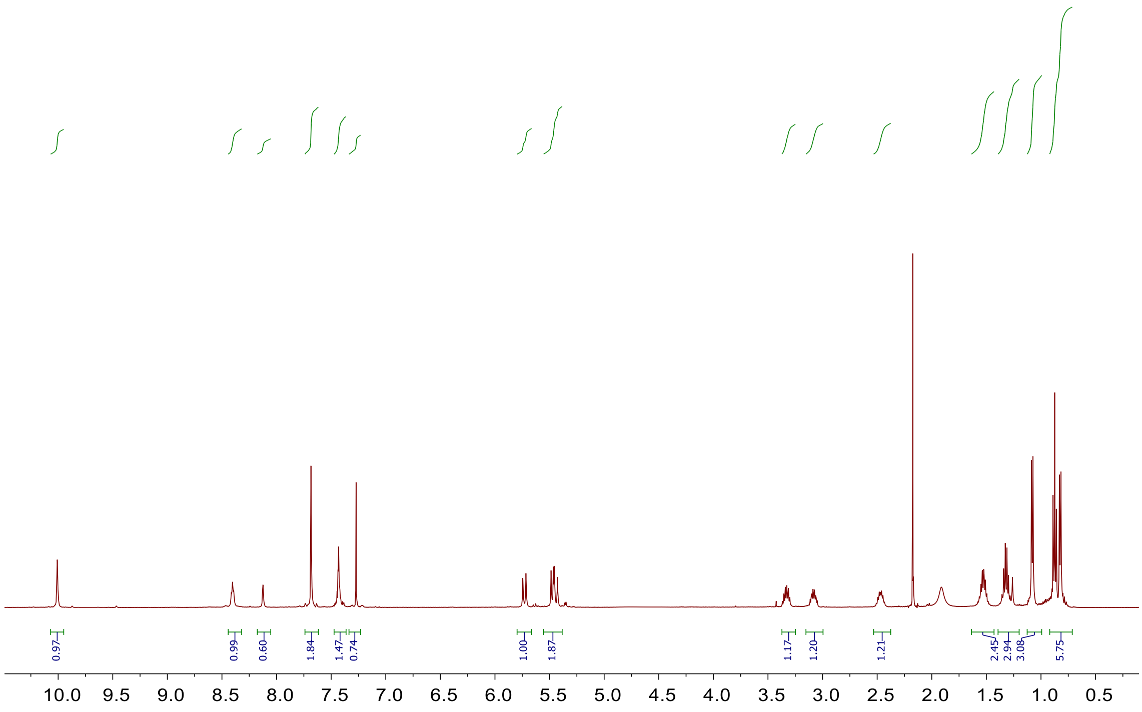

Fig. S33. ^{1}H NMR (500 MHz) of **2c** in $CDCl_3$.

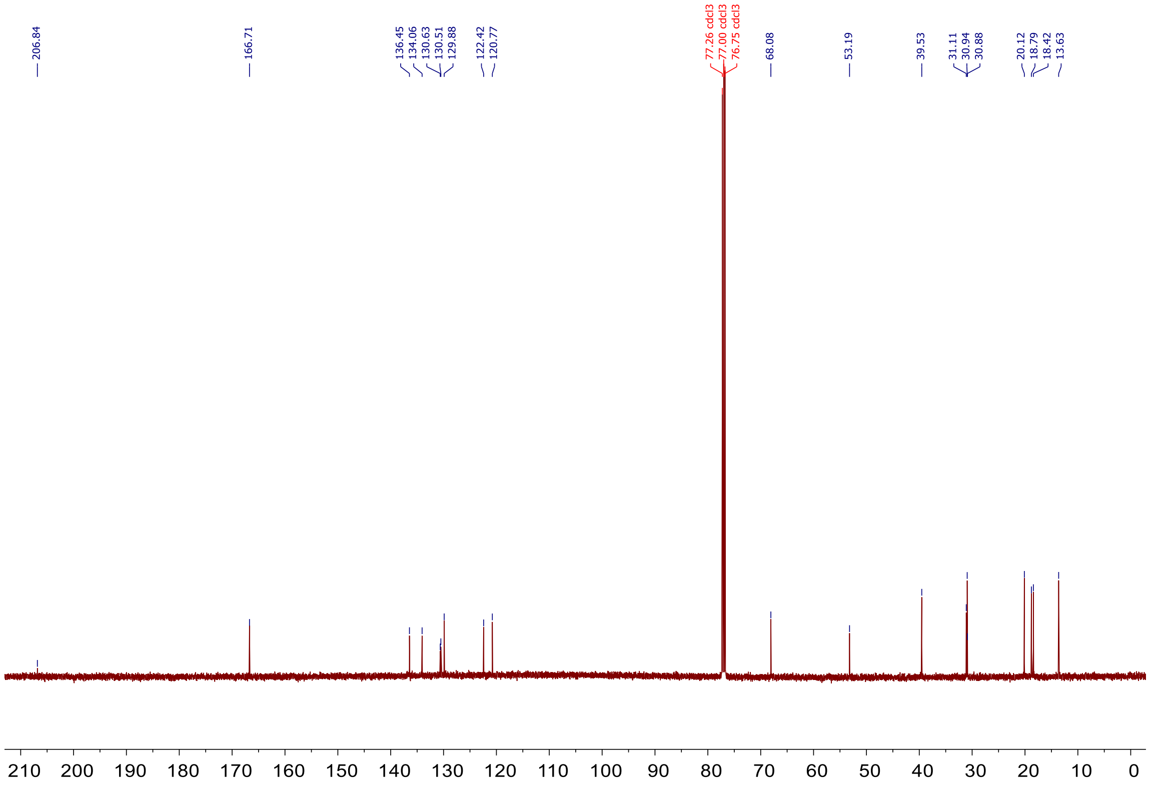

Fig. S34. ^{13}C NMR (126 MHz) of **2c** in $CDCl_3$.

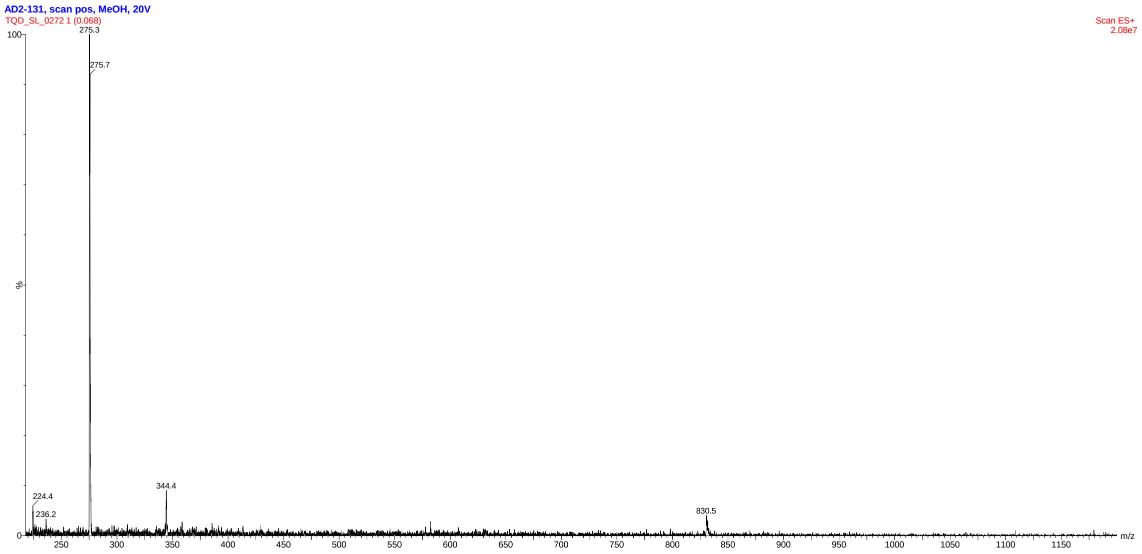

Fig. S35. MS (ESI) of **2c**.
